# Identification of hydroxy fatty acid and triacylglycerol metabolism-related genes in lesquerella through seed transcriptome analysis

**DOI:** 10.1186/s12864-015-1413-8

**Published:** 2015-03-24

**Authors:** Hyun Uk Kim, Grace Qianhong Chen

**Affiliations:** Department of Agricultural Biotechnology, National Academy of Agricultural Science, Rural Development Administration, Jeonju, 560-500 Republic of Korea; U.S. Department of Agriculture, Western Regional Research Center, Agricultural Research Service, 800 Buchanan Street, Albany, CA 94710 USA

**Keywords:** Hydroxy fatty acid, Lesquerella, *Physaria fendleri*, Seed, Transcriptome, Triacylglycerol, Gene expression, Quantitative polymerase chain reaction

## Abstract

**Background:**

Castor oil is the only commercial source of hydroxy fatty acid that has industrial value. The production of castor oil is hampered by the presence of the toxin ricin in its seed. Lesquerella seed also accumulates hydroxy fatty acid and is free of ricin, and thus it is being developed as a new crop for hydroxy fatty acid production. A high-throughput, large-scale sequencing of transcripts from developing lesquerella seeds was carried out by 454 pyrosequencing to generate a database for quality improvement of seed oil and other agronomic traits. Deep mining and characterization of acyl-lipid genes were conducted to uncover candidate genes for further studies of mechanisms underlying hydroxy fatty acid and seed oil synthesis.

**Results:**

A total of 651 megabases of raw sequences from an mRNA sample of developing seeds was acquired. Bioinformatic analysis of these sequences revealed 59,914 transcripts representing 26,995 unique genes that include nearly all known seed expressed genes. Based on sequence similarity with known plant proteins, about 74% (19,861) genes matched with annotated coding genes. Among them, 95% (18,868) showed highest sequence homology with Arabidopsis genes, which will allow translation of genomics and genetics findings from Arabidopsis to lesquerella. Using Arabidopsis acyl-lipid genes as queries, we searched the transcriptome assembly and identified 615 lesquerella genes involved in all known pathways of acyl-lipid metabolism. Further deep mining the transcriptome assembly led to identification of almost all lesquerella genes involved in fatty acid and triacylglycerol synthesis. Moreover, we characterized the spatial and temporal expression profiles of 15 key genes using the quantitative PCR assay.

**Conclusions:**

We have built a lesquerella seed transcriptome that provides a valuable reference in addition to the castor database for discovering genes involved in the synthesis of triacylglycerols enriched with hydroxy fatty acids. The information obtained from data mining and gene expression profiling will provide a resource not only for the study of hydroxy fatty acid metabolism, but also for the biotechnological production of hydroxy fatty acids in existing oilseed crops.

**Electronic supplementary material:**

The online version of this article (doi:10.1186/s12864-015-1413-8) contains supplementary material, which is available to authorized users.

## Background

Lesquerella [*Physaria fendleri*, formerly *Lesquerella fendleri* (Gray) Wats.] [1], is a potential Brassicaceae oilseed crop for the southwest region of the United States. The seed oil of lesquerella is rich in lesquerolic acid (14-hydroxy-eicos-*cis*-11-enoic acid: 20:1-OH), a hydroxy fatty acid (HFA) comprising 55-60% of total seed fatty acids [2-6]. The conventional source of HFA is castor (*Ricinus communis*) seeds; 90% of castor oil is ricinoleic acid (12-hydroxy-octadec-*cis*-9-enoic acid: 18:1-OH). Ricinoleic acid and its derivatives are used as raw materials for numerous industrial products, such as lubricants, plastics and surfactants [7]. The production of castor oil, however, is hampered by the presence of the toxin ricin and hyper-allergic 2S albumins in its seed. Lesquerella on the other hand, does not have such biologically toxic compounds, and thus its oil represents a safe source of HFA. With the development of clean and renewable energy, hydroxy fatty acid methyl esters of lesquerella oil were found to be excellent lubricity enhancers in diesel fuels [8,9] that replace sulfur-based petroleum lubricity additives, and thus reduce environmental pollution. Besides the HFA, several co-products can be obtained from lesquerella. Seed meal after oil extraction is high in protein and the amino acid lysine and could be used as livestock feed [10,11]. Gums from the seed coat and seed meal could be used as thickening or gelling agents in food and pharmaceutical products [12-15].

Considerable efforts have been made to improve the agronomics of lesquerella through plant breeding [2,16-19]. Furthermore, stable genetic transformation has been established in lesquerella [20], which provides means to quickly improve this crop through genetic engineering. Currently, the United States Department of Agriculture (USDA) National Plant Germplasm System (NPGS) has a *Phyasaria* germplasm collection of over 212 accessions representing 32 species. Variation in fatty acids among species was reported. In species *P. lindheimeri* and *P. pallida*, 20:1-OH was the most abundant, comprising over 80% in seed oil [21]. Some species have seeds with oil rich in other HFAs, such as densipolic acid (12-hydroxy-octadec-cis-9,15-enoic acid: 18:2OH) in *P. perforata*, *P. stonensis*, *P. densipila*, *P. lyrata*, and *P. lescurii* (average over 40%) [21-23]. In species *P. auriculata* and *P. densiflora*, auricolic acid (14-hydroxyeicos-cis-11,17-enoic acid: 20:2-OH) was the prevalent HFA, at 34-40% levels [24,25]. These species with different HFA profiles are valuable genetic resource and may contribute to the improvement of lesquerella cultivars.

Seed oil is stored as triacylglycerol (TAG). Biosynthesis of TAG in lesquerella follows the pathways for fatty acid (FA) in the plastid and TAG in the endoplasmic reticulum (ER) [26,27]. After the FAs are synthesized in the plastid (mostly oleic acid 18:1 with small amounts of palmitic acid 16:0 and stearic acid 18:0), they are released and then converted to acyl-Co-enzyme A (CoA). The newly synthesized acyl-CoAs can be incorporated into TAG through the glycerol-3-phosphate (G3P) pathway also known as the Kennedy pathway [28,29]. Briefly, G3P is first acylated by glycerol-3-phosphate acyltransferase (GPAT), followed by a second acylation by the acyl-CoA:acylglycerol-3-phosphate acyltransferase (LPAT), yielding phosphatidic acid (PA). PA is then hydrolyzed to form diacylglycerol (DAG), which is finally used as a substrate for the diacylglycerol acyltranstransferase (DGAT) to produce TAG. The newly synthesized acyl-CoAs can also be incorporated directly into membrane lipid phospatidylcholine (PC) by the acyl editing reactions or Lands cycle [30-32]. These acyl editing reactions can be catalyzed either by forward and reverse reactions of lyso-PC acyltransferase (LPCAT) to yield acyl-CoA, or by a phospholipase A–type activity to yield a free FA that then is activated to acyl-CoA. Since PC is the substrate for many FA-modifying enzymes (desaturase, hydroxylase, etc.), rapid de-acylation and re-acylation of PC results in an acyl-CoA pool enriched with modified FAs, which are then utilized for TAG synthesis [33,34]. Additionally, accumulating evidence indicates that many plants utilize PC-derived DAG to synthesize TAG. The main PC to DAG conversion is catalyzed by phosphatidylcholine:diacylglycerol cholinephosphotransferase (PDCT) through the phosphocholine headgroup exchange between PC and DAG [35,36]. Thus acyl editing and PC-DAG interconversion through LPCAT and PDCT, respectively, may co-contribute to the formation of TAGs with enriched modified FAs. Besides, TAG synthesis is not as simple as the sequential acylation of glycerol with GPAT, LPAT, and DGAT by the Kennedy pathway. The enzyme Phospholipid:DAG acyltransferase (PDAT) also syntheses TAG by transacylation of the *sn-2* FA from PC onto *sn-3* position of DAG, with lyso-PC as a co-product [37].

The molecular and biochemical bases of HFA synthesis have been investigated mostly in castor, lesquerella, and Arabidopsis (review) [38]. Based on studies in castor, 18:1-OH is formed by the hydroxylation of 18:1 esterified to the *sn*-2 position of PC [39,40]. Then, the 18:1-OH is released from 18:1-OH-PC and activated to 18:1-OH-CoA. In lesquerella, due to an efficient microsomal elongation system, newly formed 18:1-OH-CoA is elongated to 20:1-OH-CoA [24,25,41]. Genes encoding the oleate 12-hydroxylase (*FAH*) have been isolated from castor (*RcFAH12*) [42] and lesquerella (*PfFAH12*) [43]. (*LFAH12* or *LfFAH12* were used in publications before this report). Arabidopsis is a model oilseed that usually does not produce HFA. Expression of the *RcFAH12* in Arabidopsis leading to HFA accumulation thus demonstrated that this enzyme is directly responsible for synthesis of 18:1-OH [42,44]. Expression of *PfFAH12* in Arabidopsis [43,44] and yeast [45] has revealed that the lesquerella enzyme is bifunctional and can catalyze ∆12 hydroxylation to produce 18:1-OH and ∆12 desaturation to produce 18:2. In lesquerella, a gene encoding a condensing enzyme, *PfKCS18* (*LfKCS3* was used in publications before this report) has been isolated, and its activity has been shown to specifically catalyze elongation of 18:1-OH-CoA [41]. Besides 18:1-OH and 20:1-OH, lesquerella seed accumulates a low level of 20:2-OH, which is formed by a microsomal ∆15 desaturase [24,25,44].

Although enzymatic reactions and key genes involved in the HFA synthesis have been elucidated, mechanisms contributing to the accumulation of HFA in TAG are largely unknown. Transgenic experiments have consistently failed to achieve high yields of desired HFAs. Seed-specific expression of *RcFAH12* in Arabidopsis resulted in HFA accumulation at 17% of total seed lipids [44,46-48], which is much lower than 90% level of 18:1-OH in castor seeds. Efforts have been made to search for additional genes, especially those involved in a final step of TAG synthesis. It was shown that co-expression of a second gene, *RcDGAT2* [49] or a *RcPDAT* [50,51] boosted HFA content from 17% to nearly 30% or 25-27%, respectively. When triple transgenic Arabidopsis (carrying *RcFAH*, *RcDGAT2* and *RcPDAT1A*) is compared with a double transgenic line (carrying *RcFAH* and *RcPDAT1A*), HFA increased slightly from 25.4% to 26.7% [50,51]. With the discovery of PDCT, a castor gene *RcPDCT* was co-expressed in the transgenic Arabidopsis line carrying *RcFAH*. It indeed increased HFA from 17% to 23% in Arabidopsis [35]. Additional expression of *RcDGAT2* further enhanced the HFA content to 28% [35].

Broadening our knowledge on HFA-containing TAG biosynthesis undoubtedly requires the identification of more genes involved in HFA and TAG metabolism. The high-throughput 454 GX FLX pyrosequencing is a superior technology for transriptome analysis. It revolutionizes science by enabling users to acquire massive genome-wide data rapidly with low cost and labor. Because the method increases sequencing depth and coverage, it allows assembly of overlapping reads without a references sequence. It is particularly suitable for use in organisms whose genomic sequences are unknown. Prior to our work, there are only 71 lesquerella microsatellite sequences and ESTs in GenBank (http://www.ncbi.nlm.nih.gov/nucest/). In this study, we adopted 454 GX FLX pyrosequencing to analyze the seed transcriptome of lesquerella. We describe here identification of 26,995 unique transcripts from a total of 651 mega-base raw sequences, including transcripts for the majority of enzymes involved in lipid biosynthesis and metabolism. We further characterize the expression profiles of 15 key lipid genes in various tissues of lesquerella, including developing seeds, leaf, stem, root, and flower buds using quantitative PCR (qPCR) assays. Our results provide information on key target genes that can be useful in the design of future studies involving manipulation of HFA production in plants.

## Results and discussion

### The transcriptome represents a major source for lesquerella seed genes

To obtain a comprehensive profile of the transcriptome of lesquerella seeds, selecting a sample at an optimal stage during seed development is critical. According to our previous studies in lesquerella, the entire course of seed development took about 49 days after pollination (DAP) [52]. When developing seeds entered mid-maturation stages, 28-35 DAP, storage lipids, proteins, and other components of dry weights accumulated at maximum rates. Based on analysis of HFA accumulation and gene expression [5], we observed rapid synthesis and accumulation of TAG and major HFA (20:1-OH) from 28 to 35 DAP. Accompanying the accumulation of 20:1-OH, transcript levels of hydroxylase gene, *PfFAH12,* and elongase gene, *PfKCS18,* also increased steadily [5]. The collective data suggest that seeds at 28-35 DAP are enriched in the transcripts for enzymes involved in the synthesis and accumulation of 20:1-OH. Therefore, we determined that 30 DAP is a suitable time point for seed transcriptomic analyses. By twice deep sequencing using a cDNA library prepared from developing lesquerella seeds at 30 DAP, we generated a total of 1,568,943 clean reads which is equivalent to 651,314,783 bases (Table 1). Because there is no reference genome of lesquerella, de novo assembly was performed by GS De Novo Assembler (v 2.6). Among total reads, 496,246 reads were completely assembled and 109,151 reads were partially assembled to generate total 38,002 isotigs (Table 2). The average isotig length is 988 bp (Table 2), longer than the average isotig size of 828 bp observed in bitter melon seed transcriptome using Cap3 assembly software [53], 744 bp in *Ammopiptanthus monolicus* root transcriptome using Newbler [54], and 697 bp in Camelina sativa seed transcriptome using Trinity [55]. A mean isotig length of 1492 ± 899 bp was reported for camelina seed transcriptome [56]. However, this number was obtained by assembling 454 reads (mean length, 373 ± 129 bp) together with Sanger sequenced EST clones (mean length, 555 ± 169 bp) using NEWBLER v2.3 GS Assembler. About 35,573 reads not overlapping with any other reads were assembled to 21,912 singletons (Tables 1 and 2). Since our singletons were cleaned and validated with SeqClean and Lucy, the numbers of singletons and isotigs in combination indicate 59,914 total protein coding transcripts in our seed transcriptome. Overall, isotigs and singletons together were assembled from 640,970 reads which counted 41% of total reads (Table 1). About 57% of reads were identified as repeated regions (Table 1). A total of 901,748 repeat region reads were blast searched in SILVA rRNA database [57]. Among them, 863,953 reads (96%) matched rRNA sequences and 37,795 reads (4%) had no homologies. The rRNA sequences encode 28S, 26S, 18S, and 16S ribosomal RNAs. These rRNAs were probably carried over during mRNA purification process. The assembly analysis also generated 26,995 isogroups (Table 2), which represent 26,995 unique genes in the lesquerella seed transcriptome (Table 2).

**Table 1:** **Summary of sequencing reads**

Number of reads	1,568,943
Number of bases	651,314,783
Assembled reads	496,246
Partially assembled reads	109,151
Singleton	35,573
Repeat	901,748
Outlier+	15,725
Reads too short to assemble	10,500

+The read was identified by the GS De Novo Assembler as problematic.

**Table 2:** **Summary of** ***de novo*** **assembly**

	**Isogroups**	**Isotigs**	**Singletons** ^**┼**^
Number	26,995	38,002	21,912
Average contig count	1.7	2.132	
Largest contig count	2,257	16	
Number with one contig	22,649	22,994	
Average isotig count	1.4		
Largest isotig count	97		
Number with one isotig	22,698		
Number of bases (nt)		37,527,552	
Average isotig size (bp)		987.515	
N50^#^ isotig size (bp)		1,310	
Largest isotig size (bp)		11,074	

^┼^The number of valid singleton after SeqClean and Lucy.

^#^The half size of all bases reside in isotigs.

**Figure 1:**
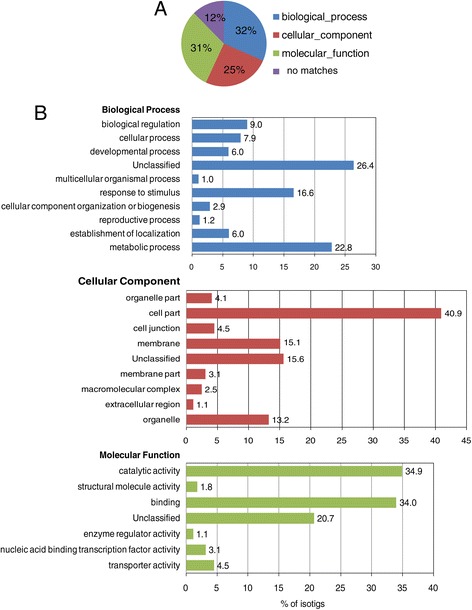
**Gene ontology (GO) annotation of all detected isotigs. (A)** Overall distribution of 38,002 isotigs into major GO categories. **(B)** The histogram shows the percentage (x axis) of isotigs within functional subdivisions (y axis) of each of the three major GO categories.

**Figure 2:**
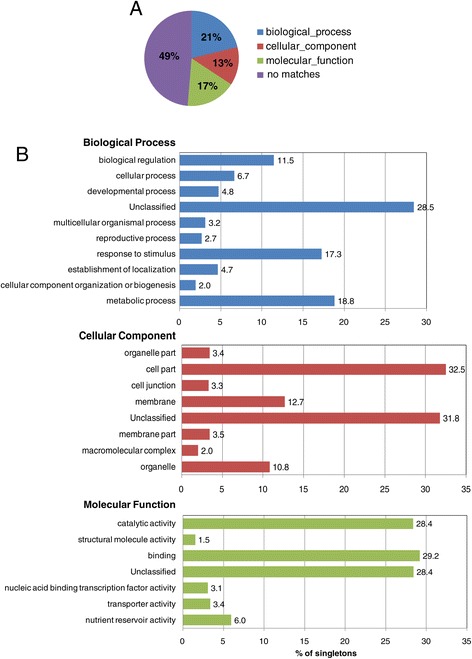
**Gene ontology (GO) annotation of all detected singletons. (A)** Overall distribution of 21,912 singletons into major GO categories. **(B)** The histogram shows the percentage (x axis) of isotigs within functional subdivisions (y axis) of each of the three major GO categories.

To functionally categorize lesquerella isotigs and singletons, we performed Gene ontology (GO) analysis. The isotigs and singletons were searched against NCBI non-redundant protein database (NR), using the Blastx program with an E-value cut-off of 1e^-3^. Among 38,002 isotigs and 21,912 singletons, 33,313 isotigs (88%) and 11,247singletons (51%) had at least one match to known protein sequences in the NCBI NR database. These isotigs or singletons fell mainly into three GO categories: biological process (11,977, 32% for isotigs; 4,641, 21% for singletons), cellular component (9,648, 25% for isotigs; 2,850, 13% for singletons), and molecular function (11,687, 31% for isotigs; 3,756, 17% for singletons). The remaining 4,689 (12%) isotigs and 10,663 (49%) singletons were not assigned (Figures 1A, 2A). In the category of “biological process”, a substantial percent of isotigs (22.8%) (Figure 1B) and singletons (18.8%) (Figure 2B) were classified in sub-category ‘metabolic process’. In category “cellular component”, ‘cell part’ had a largest percent of isotigs (26.4%) (Figure 1B) and singletons (28.5%) (Figure 2B). Within the category of “molecular function”, ‘catalytic activity’ and ‘binding’ are the two top sub-categories, with 34.9% and 34.0% respectively for isotigs, (Figure 1B), and 28.4% and 29% respectively for singletons (Figure 2B). As indicated above, the assemble analysis also generated 26,995 isogroups or unique genes. When these unique genes were searched against NCBI NR database, 19,861 isogroups (74%) matched annotated coding genes, whereas 3,134 found no homologous sequences. Besides, about 18,868 (95%) of the 19,861 isogroups with matches to known protein coding sequences had the highest homology to genes from Arabidopsis, a model dicot plant. Table 3 lists the top 50 most highly expressed genes represented by isogroups in the lesquerella seed transcriptome and their corresponding gene products based Arabidopsis orthologs. Among them, genes involved in lipid biosynthesis and metabolism were detected. These included genes encoding fatty acid desaturase 3 (FAD3), 3-ketoacyl-CoA synthase (PfKCS18), acyl-activating enzyme 17 (AAE17), hydroxysteroid dehydrogenase 5 (HSD5) and oleosins. Overall, these results demonstrate that the transcriptome data presented here provide comprehensive representation of expressed genes in developing lesquerella seed. These annotations provide a major new resource for investigating specific processes, structures, functions, and pathways that will guide research on lesquerella. Moreover, the transcriptome analysis indicates that lesquerella is closely related to Arabidopsis, which allows for translational research for seed quality enhancement in lesquerella.

**Table 3:** **List of gene products for the 50 most abundant isotigs**

**Isogroup**	**# of istotigs**	**Gene products**	**Gene ID**
isogroup00026	97	HSD5 (hydroxysteroid dehydrogenase 5)	AT4G10020
isogroup00055	64	ATAILP1	AT5G19140
isogroup00045	55	OLEOSIN 1	AT4G25140
isogroup00062	49	GAMMA-VPE, vacuolar-processing	AT4G32940
isogroup00056	46	FDH (formate dehydrogenase)	AT5G14780
isogroup00010	44	FAD3	AT2G29980
isogroup00025	41	*Physaria fendleri* 3-ketoacyl-CoA synthase	AAK62348.1
isogroup00041	37	PRXR1	AT4G21960
isogroup00046	36	HSC70-1	AT5G02500
isogroup00048	36	OLEOSIN 4	AT3G27660
isogroup00071	36	unknown protein	AT1G62220
isogroup00037	35	CESA3 (cellulose synthase 3)	AT5G05170
isogroup00047	35	Class-II DAHP synthetase family	AT1G22410
isogroup00072	35	UAP56B (homolog of human UAP56 B)	AT5G11200
isogroup00073	35	UBQ1 (ubiquitin extension protein 1)	AT3G52590
isogroup00042	34	ATNTT2	AT1G15500
isogroup00079	34	splicing factor PWI domain-containing protein	AT1G60200
isogroup00001	32	12S seed storage protein CRU4	AT5G44120
isogroup00049	32	FDM1 (factor of DNA methylation 1)	AT1G15910
isogroup00050	32	XYP1 (xylogen protein 1)	AT5G64080
isogroup00043	31	HAI1 (highly ABA-induced PP2C gene 1)	AT5G59220
isogroup00063	31	RING-type Zinc finger protein	AT5G25560
isogroup00023	30	ALPHA-TIP (alpha-tonoplast intrinsic protein)	AT1G73190
isogroup00057	30	ACO2 (ACC oxidase 2)	AT1G62380
isogroup00086	30	CESA1 (cellulose synthase 1)	AT4G32410
isogroup00080	26	BBD2 (bifuctional nuclease in basal defense response 2)	AT1G19660
isogroup00027	25	Heat shock protein 81-2,	AT5G56030
isogroup00030	25	BGLU37 (beta glucosidase 37)	AT5G25980
isogroup00044	24	NLP4 (nin-like protein 4)	AT1G20640
isogroup00058	24	TUA6 (tubulin alpha-6)	AT4G14960
isogroup00074	23	Pyruvate kinase family	AT5G08570
isogroup00038	22	UBQ1 (ubiquitin extension protein 1)	AT3G52590
isogroup00087	22	PEPR1 (PEP1 receptor 1)	AT1G73080
isogroup00088	22	Methionine synthesis 1	AT5G17920
isogroup00033	21	GTP binding Elongation factor Tu family	AT5G60390
isogroup00075	21	TCTP (translationally controlled tumor protein)	AT3G16640
isogroup00081	20	DRP2B (dynamin related protein 2B)	AT1G59610
isogroup00089	20	AOAT2 (alanine-2-oxoglutarate aminotransferase 2)	AT1G70580
Isogroup00011	19	2S seed storage protein 3	AT4g27160
isogroup00090	19	Papain family cysteine protease	AT4G16190
isogroup00064	17	Phosphoinositide phosphatase family	AT1G17340
isogroup00065	17	ALAAT1(alanine aminotransferase 1)	AT1G17290
isogroup00040	16	GTP binding Elongation factor Tu family protein	AT5G60390
isogroup00066	16	Leucine-rich repeat (LRR) family protein	AT5G45510
isogroup00083	16	HSP70 (heat shock protein 70)	AT3G12580
isogroup00067	15	BGLU37 ( beta glucosidase 37) ,	AT5G25980
isogroup00068	15	GTP binding Elongation factor Tu family	AT5G60390
isogroup00078	15	F-box/RNI-like superfamily protein	AT4G15475
isogroup00053	14	Copper amine oxidase family	AT4G12290
isogroup00061	13	AAE17(acyl-activating enzyme 17)	AT5G23050

### The transcriptome covers a broad spectrum of genes involved in acyl-lipid metabolism

Since lesquerella is a Brassicaceae oilseed crop, we used the acyl-lipid metabolism database [58] developed for model Brassicaceae oilseed Arabidopsis [31] to investigate lesquerella genes involved in acyl-lipid metabolism. We queried the transcriptome assembly to predict lesquerella orthologs of Arabidopsis lipid metabolic pathway components using reciprocal best-hits (RBH) blast approach. A total of 1,066 Arabidopsis genes from 16 sub acyl-lipid metabolism group from the lipid metabolism database [58] were used to query the lesquerella transcriptome using local blastN. Although our lesquerella genes were limited to developing seed, whereas the acyl-lipid metabolism database includes whole genome-wide acyl-lipid genes, we are able to detect total 615 lesquerella genes representing each sub acyl-lipid metabolism group (Table 4). Two subgroups, Fatty acid synthesis and Triacylglycerol biosynthesis, of lesquerella genes had 29 and 59 members, respectively, which are very close to the numbers of 28 and 69 respectively of Arabidopsis genes obtained from a microarray study on genes expressed in seeds [59]. A total of 1,989 isotigs and singletons involved in acyl-lipid metabolism showing high sequence identity with Arabidopsis genes were identified. The average number of transcripts for each subgroup were: 8 in subgroup of triacylglycerol biosynthesis and 4 in subgroup of fatty acid synthesis. The rest of subgroups each had 2-3 average transcript numbers. The result indicates that our seed transcriptome is highly representative of transcripts for fatty acid and TAG biosynthesis. We conclude that our selection of developing seeds at 30 DAP was suitable for studying acyl-lipid metabolism in lesquerella.

**Table 4:** **Number of genes and transcripts involved in acyl-lipid metabolism**

**Acyl-lipid metabolism**	**#of** ***At*** genes*	**#of expressed** ***Pf*** **genes in seed**	**#of detected** ***Pf*** **transcripts (isotigs and singletons)**	**#of transcripts per** ***Pf*** **gene**
Fatty acid synthesis	43	29	106	4
Fatty acid elongation, Desaturation & export from plastid	27	22	62	3
Prokaryotic galactolipid, Sulfolipid, & Phospholipid synthesis	61	39	91	2
Eukaryotic galactolipid & Sulfolipid synthesis	36	27	62	2
Triacylglycerol biosynthesis	87	59	483	8
Eukaryotic phospholipid synthesis & editing	75	53	122	2
Triacylglycerol & fatty acid degradation	61	41	119	3
Fatty acid elongation & wax biosynthesis	246	102	313	3
Sphingolipid biosynthesis	37	30	67	2
Mitochondria fatty acid & lipoic acid synthesis	18	11	33	3
Mitochondria phospholipid synthesis	24	17	34	2
Lipid trafficking	6	5	9	2
Cutin synthesis & transport	112	49	152	3
Suberin synthesis & transport	39	25	57	2
Oxylipin metabolism	69	36	66	2
Phospholipid signaling	125	70	213	3
Total	1066	615	1989	3

*Number of genes obtained from Arabidopsis acyl-lipid metabolism database [58].

### Genes involved in fatty acid and TAG biosynthesis are well-represented in the transcriptome

**Figure 3:**
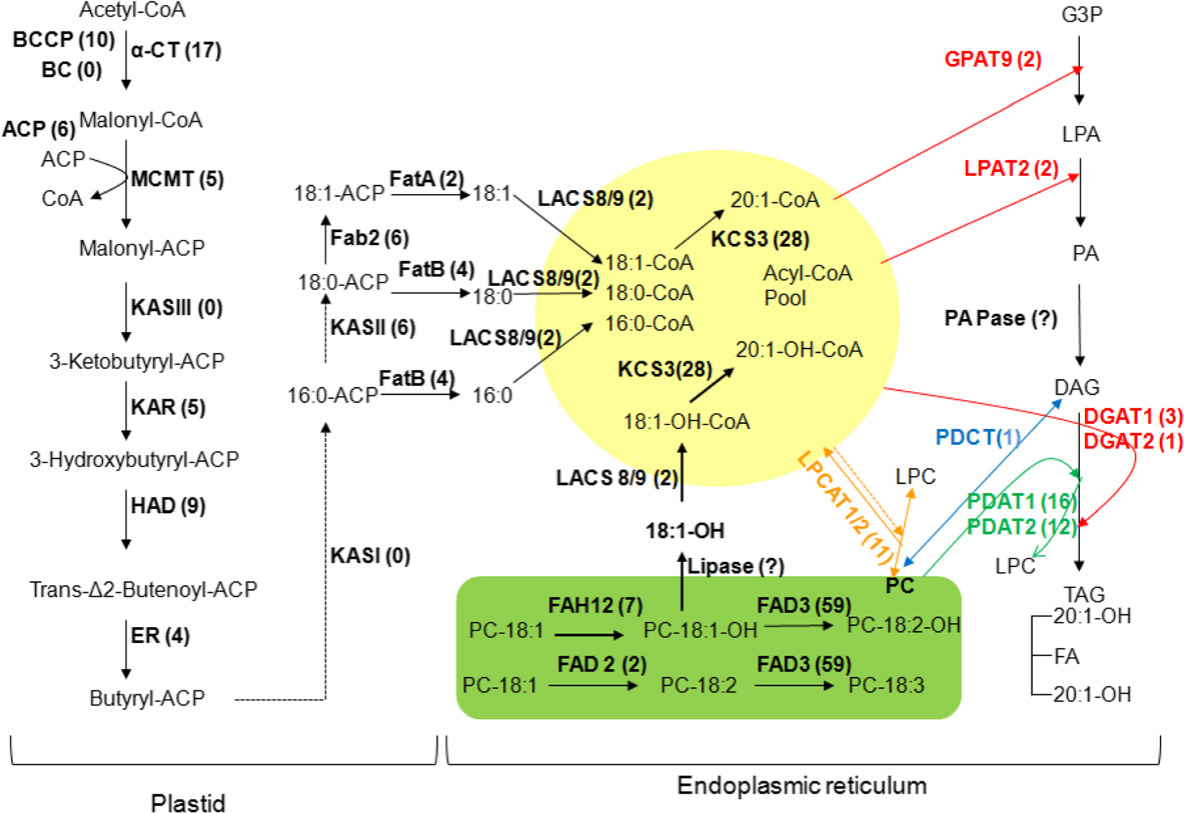
**Predicted fatty acid and TAG biosynthetic pathways in** ***P. fendleri*** **seeds.** Numbers after the gene are the numbers of isotigs for the corresponding genes in the 454 analysis of the seed cDNA library. The dashed arrows in the plastid fatty acid biosynthetic pathway indicate one or more cycles of acyl-chain elongation that is initiated by 3-ketoacyl-ACP synthase (KAS) I or II. The yellow circle delineates reactions in acyl-CoA pools. The bright green rectangle delineates reactions involving desaturation or hydroxylation of PC. The acyl-CoA dependent Kennedy pathway is indicated with red arrows. PC-mediated TAG synthesis pathways are indicated with green (via PDAT), blue (via PDCT) and orange (via LPCAT) arrows. Abbreviations: BCCP, biotin carboxyl carrier protein subunit of acetyl-CoA carboxylase; BC, biotin carboxylase subunit of acetyl-CoA carboxylase; α-CT, α-carboxyltransferase subunit of acetyl-CoA carboxylase; ACP, acyl carrier protein; MCMT, malonly-CoA ACP transferase; KAR, 3-ketoacyl-ACP reductase; HAD, 3-hydroxyacyl-ACP dyhydratase; ER, 2-enoyl-ACP reductase; FAB2/SAD, stearoyl-ACP desaturase; FatA, acyl-ACP thioesterase A; FatB, acyl-ACP thioesterase B; LACS, long-chain acyl-CoA synthase; FAH12, Δ12 oleic acid hydroxylase; FAD2, Δ12 oleic acid desaturase; FAD3, Δ15 (ω-3) linoleic acid desaturase; KCS3, 3-ketoacyl-CoA synthase 3; GPAT9, glycerol 3-phosphate acyltransferase 9 ; LPAT2, lysophosphatidic acid acyltransferase 2; PAPase: phosphatidic acid phosphatase; LPCAT, lysophosphatidylcholine acyltransferase 1 and 2; DGAT, diacylglycerol acyltransferase1 and 2; PDAT, phospholipid:diacylglycerol acyltransferase; G3P, glycerol-3-phosphate; LPA, lysophosphatidic acid; PA, phosphatidic acid; LPC, lysophosphatidylcholine; PC, phosphatidylcholine; DAG, diacylglycerol; TAG, triacylglycerol.

To understand hydroxy fatty acid biosynthesis and metabolism in lesquerella, we further deeply mined the transcriptome assembly for genes involved in fatty acid and TAG biosynthesis. Using known Arabidopsis genes listed in the acyl-lipid metabolism database, we identified lesquerella orthologs for nearly all the genes involved in fatty acid and TAG biosynthesis; the number of isotigs for each gene varied from 1 to 58 (Table 5, Figure 3). Among 32 genes, 20 (63%) had a representative isotig encoding full length cDNA. The ones coding for partial cDNA sequences covered 23-97% of their full length sequences. Strikingly, we found a high percentage of sequence identity between Arabidopsis and lesquerella genes. Out of 32 genes, 20 had nucleotide identity at 90-99%, with an average identity overall of 90%. The remaining 12 had 80-89% identity (Table 5). The very high sequence identity between Arabidopsis and lesquerella genes suggests a similar high degree of conservation of their functions in seeds that enables translational research and facilitates genetic engineering of lesquerella lines with desirable oil content and fatty acid composition.

**Table 5:** **List of expressed genes involved in fatty acid and TAG biosynthesis in lesquerella seed**

	**Gene**	**At ID**	**isotigs**	**%of nucleotide identity**	**#of isotigs**	**ORF**
						**Partial (P) or Full length (F)**
*De novo* fatty acid biosynthesis and export from plastid	*BCCP1*	At5g16390	isotig19563	88	7	P
	*BCCP2*	At5g15530	isotig07553	89	3	F
	*α-CT*	At2g38040	isotig03259	90	17	F
	*MCMT*	At2g30200	isotig17972	93	5	P
	*ACP5*	At5g27200	isotig07684	89	6	F
	*KASII*	At1g74960	isotig15566	91	6	F
	*KAR*	At1g24360	isotig08993	91	5	F
	*HAD*	At5g10160	isotig23452	88	9	P
	*ER*	At2g05990	isotig17121	93	4	F
	*FAB2*	At2g43710	isotig11458	91	6	F
	*FatA-1*	At3g25110	isotig17885	89	1	F
	*FatA-2*	At3g25110	isotig17382	90	1	F
	*FatB*	At1g08510	isotig09645	88	4	P
	*LACS8*	At2g04350	isotig15424	90	1	F
	*LACS9*	At1g77590	isotig15369	89	1	P
Endoplasmic reticulum-hydroxylase, desaturase, and elongase	*FAH12*	AF016103	isotig27455	95	7	F
	*FAD2*	At3g12120	isotig17487	88	2	F
	*FAD3-1*	At2g29980	isotig17127	92	1	F
	*FAD3-2*	At2g29980	isotig00061	84	58	F
	*KCS18*	AF367052	isotig02742	97	28	F
Acyl-CoA- dependent TAG synthesis in Kennedy pathway	*GPAT9*	At5g60620	isotig18564	94	2	P
	*LPAT2*	At3g57650	isotig03872	92	2	F
	*DGAT1-1*	At2g19450	isotig11157	91	2	P
	*DGAT1-2*	At2g19450	isotig11156	90	1	P
	*DGAT2*	At3g51520	isotig19956	87	1	F
	*DGAT3*	At1g48300	isotig08903	91	3	F
PC-mediated TAG synthesis	*LPCAT1*	At1g12640	isotig03769	91	10	F
	*LPCAT2*	At1g63050	isotig16468	92	1	P
	*PDAT1-1*	At5g13640	isotig08780	92	1	F
	*PDAT1-2*	At5g13640	isotig08781	90	15	P
	*PDAT2*	At3g44830	isotig02595	86	12	P
	*PDCT*	At3g15820	isotig25038	89	1	P

#### Fatty acid biosynthesis in plastids

We examined our collection of lesquerella transcripts for representatives of key genes encoding the known steps of fatty acid biosynthesis in plastids (Figure 3). Fatty acid biosynthesis begins with the rate-limiting conversion of acetyl-CoA to malonyl-CoA by acetyl-CoA carboxylase, a heteromeric complex enzyme composed of 4 subunits: 1 beta-carboxyltransferase (β-CT) encoded by the plastid genome and biotin carboxyl-carrier protein (BCCP), biotic carboxylase (BC), and alpha-carboxyltransferase (α-CT), each encoded by the nuclear genome. In the lesquerella transcriptome, transcripts encoding BCCP and α-CT subunits were identified with 10 isotigs and 17 isotigs, respectively (Table 5). In Arabidopsis, two paralogous of *BCCP* genes, *BCCP1* (At5g16390) and *BCCP2* (At5g15530) were characterized with *BCCP1* being the more highly expressed during embryo development [60]. In lesquerella seed, 7 isotigs of *PfBCCP1* and 3 isotigs of *PfBCCP2* were identified, and the longest, isotig19563 and isotig07553, shared 88% and 89% nucleotide sequence identity, respectively, to Arabidopsis homologous. We did not detect *BC* and *β-CT* in the lesquerella seed transcriptome, which could be due to a low level of their transcripts in 30 DAP seeds. We detected lesquerella homologs of all five isoforms of plastid acyl-carrier proteins (ACP) reported in Arabidopsis (At3g05020, At1g54580, At1g54630, At4g25050, At5g27200) [61]. Among them, *PfACP5* (6 isotigs), corresponding to At5g27200 isoform, was the mostly expressed (Table 5). A gene encoding malonyl-CoA ACP transferase (MCMT) was also identified in lesquerella with 5 isotigs, and the longest isotig17972 showed 93% nucleotide sequence identify with its Arabidopsis MCMT homolog (At2g30200).

Fatty acid synthesis is continued by an acyl-chain specific condensing enzyme subunit (KASIII, I, and II), and the common component of 3-ketoacyl-ACP reductase (KAR), 3-hydroxyacyl-ACP dehydratase (HAD), and 2-enoyl-ACP reductase (ER) (Figure 3). We tried to identify transcripts for three key fatty acid synthases, 3-ketoacyl-ACP synthase (KAS) III, I, II using Arabidopsis KASIII (At1g62640), KASI (At5g46290) and KASII (At1g74960) genes as queries, but only *PfKASII* was detected. It had 6 isotigs, and the longest, isotig15566, showed 91% identity with Arabidopsis seed homolog KASII [62]. The other two KAS transcripts are apparently missing or rare in the 30-day seed transcriptome. Based on Arabidopsis KAR (At1g24360), HAD (At5g10160), and ER (At2g05990), we detected *PfKAR* (5 isotigs) , *PfHAD* (9 isotigs) and *PfER* (4 isotigs) in lesquerella seed. Stearoyl-ACP desaturase (SAD) catalyzes the conversion of 18:0-ACP to 18:1-ACP in plastids (Figure 3). Arabidopsis has seven SAD family genes including FAB2 (At2g43710) and DES5 (At1g02630); FAB2 is the most highly expressed [63]. Indeed the FAB2 plays a major role in the reaction [64]. In the seed transcriptome, lesquerella FAB2 homologues were detected with 6 isotigs; No homologous isotig was detected for DES5 (Table 5, Figure 3). Two fatty acid thioesteases, FatA (homologue of At3g25110) and FatB (homologue of At1g08510), were detected with 2 and 4 isotigs, respectively in lesquerella seed (Table 5, Figure 3). Long chain acyl-CoA synthase (LACS) is located the membrane of plastid outer envelopes and catalyzes addition of CoA to free fatty acids to produce the fatty acyl-CoA’s utilized in the endoplasmic reticulum. Two Arabidopsis plastid-localized LACS9 (At1g77590) and ER-localized LACS8 (At2g04350) have been reported [65,66]. In the lesquerella seed transcriptome, 1 isotig of *PfLACS8* and 2 isotigs of *PfLACS9* were identified (Table 5, Figure 3).

#### Endoplasmic reticulum-associated fatty acid hydroxylase, desaturases and elongase

Seed oil of lesquerella contains 55-60% 20:1-OH, and two key genes, *PfFHA12* and *PfKSC18,* directly responsible for synthesis of this unusual fatty acid have been previously identified [41,43]. In our seed transcriptome, we found 7 and 28 isotigs representing *PfFAH12* and *PfKCS18,* respectively (Table 5, Figure 3). The detailed temporal expression patterns of *PfFAH12* and *PfKCS18* during lesquerella seed development were reported [5]. Both of the genes showed a bell-shaped expression pattern with a peak at 35 DAP. The increased expression of *PfFAH12* and *PfKCS18* coincided with the increased synthesis and accumulation of HFA-containing TAG during lesquerella seed development [52].

**Figure 4:**
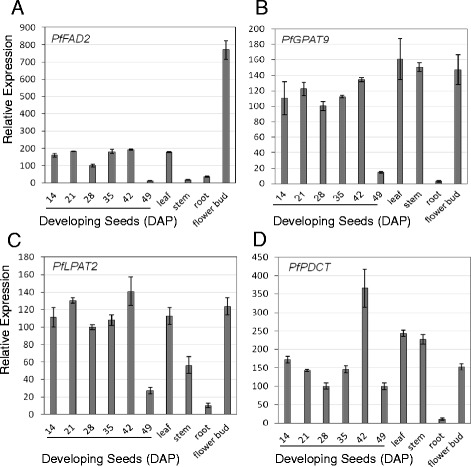
**Expression of** ***PfFAD2*** **(A),** ***PfGPAT9*** **(B),** ***PfLPAT2*** **(C) and** ***PfPDCT*** **(D) in major organs and developing seeds determined by qPCR.** Abbreviated names for the genes are defined in Figure 3. Each data point represents the mean ± SD of three replicates.

ER-associated microsomal oleoyl PC desaturase encoded by FAD2 is known to introduce a double bond at the ∆12 position of 18:1 on PC and convert it to linoleic acid (18:2) (Figure 3). During the entire course of seed development, lesquerella accumulates 18:2 at 2.5-6.5 mol% in TAG with an average of 4.8 mol% in TAG [5]. The presence of a constant level of 18:2 in lesquerella seeds indicates that an oleate desaturease activity is maintained at a relatively steady level. In this study, transcripts of *PfFAD2* desaturase were detected with 2 isotigs, fewer than those of *PfFHA12* and *PfKSC18.* The results of temporal expression profile analysis indicated that *PfFAD2* is expressed at a relatively constant level throughout most stages of seed development up to 42 DAP, but drops about 95% by the latest stage sampled, 49 DAP (Figure 4A). The temporal expression pattern of *PfFAD2* observed in this study is slightly different from that previously reported (*LfFen1*) [5], where a bell-shaped pattern of *PfFAD2* expression was observed that peaked at 35 DAP and decreased at 42 and 49 DAP [5]. We noted in that study that the change of the expression level of *PfFAD2* during seed development was moderately dynamic. In young seeds up to 21 DAP, the expression were already 36-48% of that of 28 DAP. During 28-35 DAP maturation stages, seeds boosted the expression only 2 to 4-fold [5]. The difference of *PfFAD2* expression pattern at 42 DAP in the two studies could be due to variation in seed sample groups. Nonetheless, the constitutive or less dynamic temporal expression patterns of *PfFAD2* indicated its house-keeping function for membrane lipid during seed development. Similar temporal patterns were found in some other house-keeping genes involved in seed oil biosynthesis in Arabidopsis [67] and castor [68]. In this study, we further characterized the expression profile of *PfFAD2* in leaf, stem, root and flower bud tissues (Figure 4A). We found that leaf had a level similar to that of developing seeds. The result is consistent with previously reported data of Northern blot analysis [43] or reverse transcription PCR [69]. However, a 4.4-fold higher expression of *PfFAD2* in flower than leaf was observed (Figure 4A). Since our flower buds contain about 26% pollen by weight, it is likely that *PfFAD2* plays a key role in converting 18:1 to 18:2 to meet the demand of maintaining a basic level of 18:2 in lesquerella pollen. Similar results was reported for a safflower *FAD2-10* that was expressed at a higher level in flower than cotyledon, hypocotyl, root, leaf and seed [70]. In addition, a *FAD2* from *Brassic napus* was also found to be highly expressed in flower buds, and the expression was associated with membrane lipids and storage oil biosynthesis in pollen [71]. Recently, *PfFAD2* has been shown to encode functional Δ12 desaturase activity in transformed yeast [69]. Although the bi-functional enzyme encoded by the *PfFAH12* gene also has some oleate 12-desaturase activity [43,45] that could convert some of the 18:1 to 18:2, we suggest that *PfFAD2* plays an essential role in the desaturation of 18:1 in all cells and tissues.

**Figure 5:**
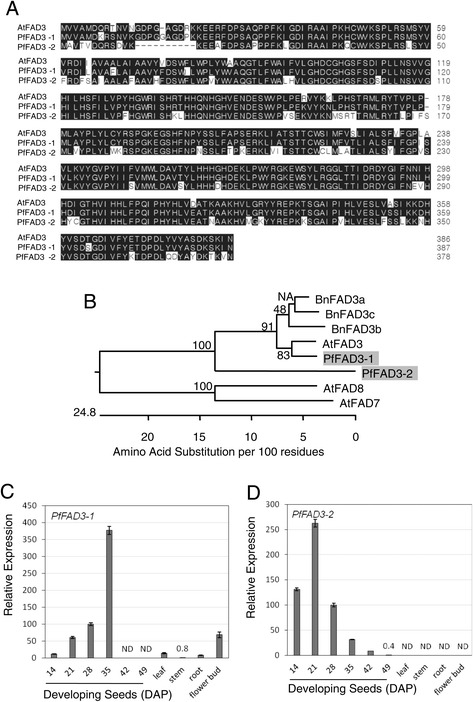
**Characterization of two** ***PfFAD3*** **. (A)** Amino acid sequences alignment among Arabidopsis FAD3 (AtFAD3:At2g29980), *PfFAD3-1* (isotig17127) and *PfFAD3-2* (isotig00061). Black shading indicates identical amino acids. No shading indicates dissimilar amino acids. Dashes indicate gaps in alignment. **(B)** Phylogenetic tree showing relationships among FAD3 and FAD7/8 desaturases. Lesquerella sequences are shaded. This tree was constructed by DNASTAR MegAlign program using the ClustalW method. Bootstrap used trials = 1000, seed = 111. At, Arabidopsis, Bn, *Brassica napus* FAD3a (AFJ19040), FAD3b (AFJ19037) and FAD3c (AFJ19034). Arabidopsis FAD7 (At3g11170) and FAD8 (At5g05580) used for outgroup. Expression of *PfFAD3-1* **(C)** and *PfFAD3-2* **(D)** in developing seeds and major organs determined by qPCR. Abbreviated names for the genes are described in Figure 3. Each data point represents the mean ± SD of three replicates. ND = not detected.

Interestingly *FAD3* transcripts were detected with the highest number (59 isotigs) among all genes involved in fatty acid and TAG biosynthesis (Table 5). These FAD3 may catalyze PC-18:2 to PC-18:3 and/or PC-18:1-OH to PC-18:2-OH in lesquerella (Figure 3). Result of amino acid alignment of the isotigs indicated that there are two FAD3 isoforms in lesquerella compared to single *FAD3* gene in Arabidopsis (Figure 5A, 5B). *PfFAD3-1,* represented by isotig17127, had 93-95% identity to Arabidopsis *AtFAD3* and *Brassica napus BnFAD3a, BnFAD3b, and BnFAD3c* [71], indicating a common FAD3 in Brassicaceae plants. However, *PfFAD3-2,* represented by isotig00061, is an isoform diverged from all the above *FAD3s,* with only 78-81% identity to *AtFAD3* and *BnFDA3s* (Figure 5B). Isotig17127 and isotig00061 showed distinct N-terminal coding regions (Figure 5A). Our expression profiling studies indicate that the expression of *PfFAD3-1* progressively increased during seed development reaching a peak at 35 DAP, and then dropped sharply to undetectable levels at late stages 42 and 49 DAP (Figure 5C). The induction between 14 DAP and 35 DAP was 33-fold. Moderate levels of expression were detected in leaf, stem and root. In flower bud, the expression was at about 6-fold higher than in seeds at 14 DAP (Figure 5C). The temporal and spatial expression pattern of *PfFAD3-2* is quite different from that of *PfFAD3-1* (Figure 5D). At early stages of seed development (14-21 DAP), the expression of *PfFAD3-2* was high and then declined quickly when seeds progressed to mid- and late-stages. No expression was detected in leaf, stem, root and flower buds. In our seed transcriptome (30 DAP), many more *PfFAD3-2* transcripts were detected (58 isotigs) than *LfFAD3-1* (1 isotig) (Table 5). This could be due to that the sample used for transcriptome study represented relatively young seeds. Based on the expression profiles of these two *PfFAD3* genes, we suggest that both of them may contribute to the desaturation of FA in developing seeds, but *PfFAD3-1* had a pattern similar to that of Arabidopsis (microarray data), and thus it may have a more general role in converting 18:2 to 18:3 in other organs. Since lesquerella seeds accumulate moderate amounts of 18:2-OH and 20:2-OH, the divergent and also seed-specific PfFAD3-2 isoform might have evolved to desaturate 18:1-OH and 20:1-OH. Further functional characterization of *PfFAD3-1* and *PfFAD3-2* needs to be carried out in order to determine their enzymatic activity and substrate specificity in acyl-lipid metabolism.

#### Conventional Kennedy pathway for TAG synthesis in ER

The conventional Kennedy pathway for TAG synthesis utilizes three acyl-CoA-dependent acyltransferases, GPAT, LPAT and DGAT, that sequentially acylate the *sn-*1- and *sn-*2- and then *sn-*3-position of G3P with acyl-CoA (Figure 3, red arrows). Since the synthesis of membrane glycerolipids also begins with sequential acylation of the *sn-*1- and *sn-*2- positions of G3P, GPAT and LPAT are common to synthesis of TAG and membrane glycerolipids. Using a bioinformatics approach, a new GPAT (At5g60620) was identified in Arabidopsis that exhibited extensive homology with a GPAT from mammalian cells involved in storage oil formation; that GPAT was postulated to be a putative AtGPAT9 for ER associated membrane and storage lipid biosynthesis in plants [72]. For the second acyl-CoA transferase, Arabidopsis LPAT2 (At3g57650) was found to be an ER-localized and involved in TAG and membrane lipid biosynthesis [73]. Using Arabidopsis genes (At5g60620 and At3g57650), we identified lesquerella orthologs of *PfGPAT9* and *PfLPAT2*, each represented by 2 isotigs (Table 5). Result of gene expression analysis indicated that both genes were expressed at a similar level in most samples examined, including leaf, stem, flower bud, and developing seeds from 14 DAP to 42 DAP, with the exception of *PfLPAT2* levels in stem tissue, where expression was only about 50% that detected in leaf (Figure 4B, 4C). Low levels of expression were detected in root and developing seeds at 49 DAP (Figure 4B, 4C). Our spatial and temporal expression profiles of *PfGPAT9* and *PfLPAT2* were similar to those from Arabidopsis [59,73-75]. Based on the overall spatial and temporal expression profiles of *PfGPAT9* and *PfLPAT2*, we suggest both genes playing essential housekeeping roles in membrane and storage lipid biosynthesis throughout plant life.

**Figure 6:**
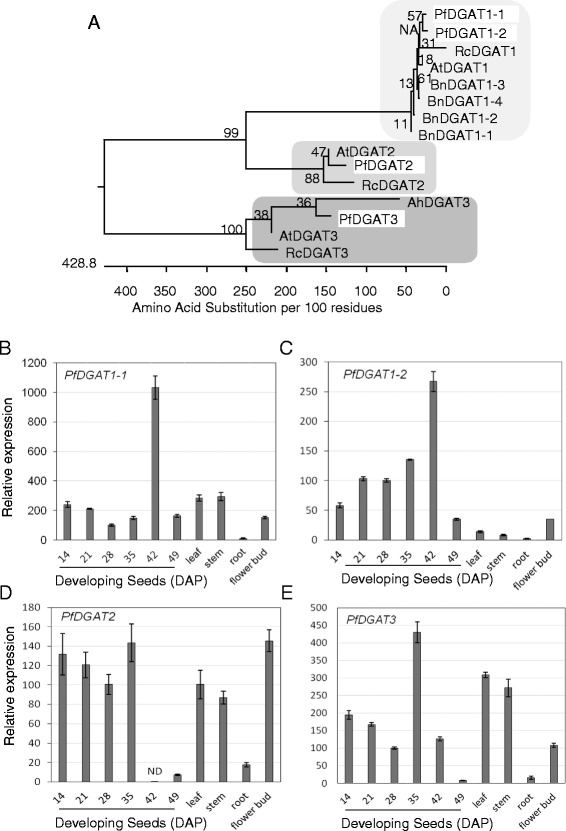
**Characterization of three types** ***PfDGATs*** **. (A)** Phylogenetic tree showing relationships among DGAT1, DGAT2, and DGAT3 from *Physaria fendleri* (Pf), Arabidopsis (At), *Brassica napus* (Bn), castor bean (Rc), peanut (Ah). *PfDGAT1-*1 (isotig11157), *PfDGAT1-*2 (isotig11156), *PfDGAT2* (isotig19956), *PfDGAT3* (isotig08903), AtDGAT1 (At2g19450), AtDGAT2 (At3g51520), AtDGAT3 (At1g48300), BnDGAT1-1 (AIA77019), BnDGAT1-2 (AIA67020), BnDGAT1-3 (AFM31260), BnDGAT1-4 (AAF64065), RcDGAT1 (XP_002514132), RcDGAT2 (XP_002528531), RcDGAT3 (XP_002519339), AhDGAT3 (AAX62735). The tree was constructed as described in Figure 5B. **(B-E)** Expression of *PfDGAT1-1, PfDGAT1-2, PfDGAT2, and PfDGAT3* in developing seeds and major organs determined by qPCR. Abbreviated names for the genes are described in Figure 3. Each data point represents the mean ± SD of three replicates.

DGAT catalyzes the final and rate-limiting step of TAG biosynthesis (Figure 3). There are three sequence-unrelated classes of DGATs reported in plants: membrane bound DGAT1 and DGAT2, and cytosolic DGAT3 (review) [76]. Using Arabidopsis *DGAT1* (At2g19450), *DGAT2* (At3g51520), and *DGAT3* (At1g48300) as queries, seven lesquerella orthologs were identified in the seed transcriptome (Table 5). Phylogenic analysis among *DGAT* genes from lesquerella, Arabidopsis, castor bean and peanut shows that the membrane type 1 (*DGAT1*), type 2 (*DGAT2*) and type 3 (*DGAT3*) are divided into three different clades (Figure 6A). The result of amino acid alignment of the isotigs indicates that there are two *PfDGAT1* isoforms in lesquerella compared to single *DGAT1* gene in Arabidopsis (Figure 6A, Additional file 1: Figure S1). Isotig11156 and isotig11157 had overall similarities in nucleotide and amino acid (AA) sequences (Table 5), but the first 100 AA at the N-termini show 45% divergence for isotig11156 and 53% for isotig11157 (Additional file 1: Figure S1). We designate the isoform encoded by isotig11157 as *PfDGAT1-1* and that encoded by isotig11156 as *PfDGAT1-2*. Lesquerella *DGAT2* is more related to the *DGAT2* from Arabidopsis than to that from castor (Figure 6A). Protein sequence alignment revealed that castor DGAT2 has additional 25 AA at its N-terminus (Additional file 1: Figure S2). Soluble DGAT3 is divided into a distinct clade in the phylogeny tree from *DGAT1* and *DGAT2* due to sequence distance (Figure 6A). *PfDGAT3* encodes a longer protein (360 AA) compared with those of 285, 332, and 345 AA of Arabidopsis, castor bean, and peanut *DGAT3*, respectively (Additional file 1: Figure S3). Protein sequence analysis showed that PfDGAT3 had 82% identity with AtDGAT3, even though it has 75 more AA (Additional file 1: Figure S3).

It is general accepted that depending on the plant species, DGAT1 or DGAT2 is a major enzyme responsible for the accumulation of seed TAG [76]. DGAT3 was recently demonstrated to be active in recycling of 18:2 and 18:3 FAs into TAG through a cytosolic pathway in peanut [77]. Our results of gene expression analysis showed that *PfDGAT1-1* and *PfDGAT1-2* had distinct expression patterns. *PfDGAT1-1* was expressed in all stages during seed development and in leaf, stem, and flower bud, but it was expressed more in leaf, stem, and in immature seeds prior to active oil biosynthesis and became a predominant *DGAT* mRNA at late-maturation/desiccation stages (42-49 DAP) (Figure 6B). In contrast, *PfDGAT1-2* had expression levels higher in developing seeds than in other tissues such as leaf, stem, root and flower buds (Figure 6C). *PfDGAT1-2* may specifically contribute to TAG synthesis in seed. Indeed, our *PfDGAT1-2* is the same gene as *PfDGAT1a* identified in a lesquerella seed cDNA library [78] and found to complement the Arabidopsis AS11 mutant [79]. AS11 had reduced DGAT activity and seed oil content due to a deletion in *AtDGAT1* gene [79-81]. Seed-specific over-expression of an Arabidopsis cDNA encoding *AtDGAT1* not only restored the oil content in AS11 but also enhanced seed oil content and seed weight in wild-type plants [82]. The expression profile of *PfDGAT2* was overall similar to that of *PfDGAT1-1*, except in the late-maturation/desiccation stages where *PfDGAT2* expression dropped to undetectable levels or trace amounts (42 and 49 DAP, respectively) (Figure 6D). The results indicate that both *PfDGAT1-1* and *PfDGAT2* may function in other physiological processes besides seed oil synthesis, and that they clearly contribute differently in lipid metabolism during late-maturation/desiccation stages of seed development. *PfDGAT3* was ubiquitously expressed in all samples and showed a moderate dynamic pattern compared with the other *PfDGATs*. In leaf, stem, flower bud, and developing seeds at early stages (14-21 DAP), *PfDGAT3* transcripts were detected at levels similar to that of *PfDGAT1-1* (Figure 6B, 6E). In developing seeds at 35 DAP, their levels rose 2- to 4-fold before declining steadily at late stages 42-49 DAP (Figure 6B, 6E). The boosted expression of PfDGAT3 may be associated with increasing demands of membrane and storage lipids synthesis at 35 DAP, when seeds had attained their maximum size and storage compounds have accumulated to a high plateau [52]. The temporal and spatial expression pattern of *PfDGAT3* suggests its role of house-keeping in most organs of lesquerella. Similar expression profile was reported for DGAT3 in peanut [83], Arbidopsis [75] and tung tree [84]. None of these DGAT3s were hypothesized to play a significant role in seed oil synthesis; rather it was proposed that they are involved in general TAG metabolism. Among all samples, root tissue had the lowest number of transcripts of all *PfDGATs*. While it is clear that *PfDGAT1-2* plays a role in seed TAG assembly, it remains an open question as to whether or not *PfDGAT1-1*, *PfDGAT2* or *PfDGAT3* also contribute. Measurements of enzyme activity and substrate specificity in various tissues are needed to better elucidate the functions of the different PfDGATs. The results of such studies combined with our sequence characterization and expression profiling will provide the molecular basis for future identification of *PfDGAT* candidates for genetic engineering oilseeds for hydroxy fatty acid production.

#### PC-mediated TAG synthesis

As described, PC is the substrate for many FA-modifying enzymes (desaturase, hydroxylase, etc.). The FA fluxes into and out of PC are crucial for the production of TAG esterified with modified FAs, such as HFA. Based on current knowledge, there are three routes allowing PC-derived FA to be incorporated into TAG. First, The FA esterified to PC undergoes constant deacylation and reacylation by LPCAT in so called acyl editing [27]. Thus modified FA released by LPCAT can enter Kennedy pathway for TAG assembly (Figure 3, orange arrows). Second, direct transfer by PDAT of a FA from the *sn*-2 position of PC to the *sn*-3 position of DAG produces TAG (Figure 3, green arrows). Third, PDCT catalyzes the inter-conversion between DAG and PC by phosphocholine head group exchange (Figure 3, blue arrows). Thus FA on PC can be incorporated into the *sn*-1 and *sn*-2 positions of TAG by the PC derived DAG.

**Figure 7:**
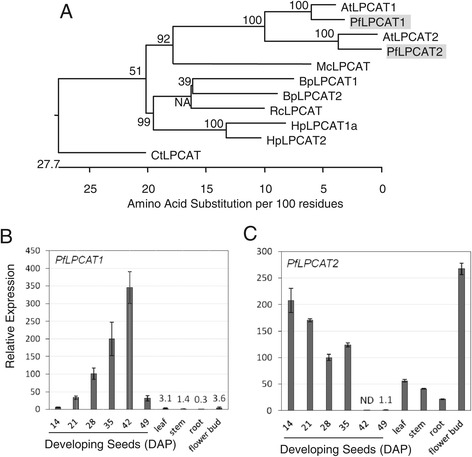
**Characterization of two** ***PfLPCATs.*** **(A)** Phylogenetic tree showing relationships among LPCAT1 and LPCAT2 from *Physaria fendleri* (Pf)*,* Arabidopsis (At)*, Bernardia pulchella* (Bp), castor bean (Rc), *Hiptage benghalensis* (Hb), *Momordica charantia* (Mc), *Carthamus tinctorius* (Ct). *PfLPCAT1* (isotig03769), *PfLPCAT2* (isotig16468), BpLPCAT1 (AHE80984), BpLPCAT2 (AHE80985), RcLPCAT (AHF20951), HpLPCAT1a (AHE80986), HpLPCAT2 (AHE80988), McLPCAT (AGL81301), CtLPCAT (AHE80989). The tree was constructed as described in Figure 5B. **(B, C)** Expression of *PfLPCAT1 and PfLPCAT2* in developing seeds and major organs determined by qPCR*.* Abbreviated names for the genes are described in Figure 3. Each data point represents the mean ± SD of three replicates.

Recent studies have revealed the roles of the enzymes encoded by Arabidopsis *LPCAT1* (At1g12640) and *LPCAT2* (At1g63050) and other plant *LPCATs* genes [85-88]. They suggest that LPCATs are responsible for incorporation of newly synthesized fatty acid into PC (forward reaction), and transferring polyunsaturated and HFAs produced on PC directly to the acyl-CoA pool (reverse reaction) for further metabolism or catabolism. When reverse reactions were measured, a ricinoleoyl group at the *sn*-2 position of PC was removed 3- to 6-fold faster than an oleoyl group by seven LPCATs from five species tested, including a *PfLPCAT2* [88]. These results suggest an important role for PfLPCATs in removing 18:1-OH from *sn*-2 PC to an acyl-CoA pool. The mechanism of PC-modified UFAs channeled into TAG through efficient acyl editing and Kennedy pathway has been proposed as one of the major mechanisms that allow efficient assembling of UFA into TAGs [32]. As lesquerella TAGs contains 55-60% 20:1-OH, it is likely that the acyl editing by PfLPCATs allows 18:1-OH to be immediately released from PC, activated to 18:1-OH-CoA then elongated to 20:1-OH-CoA for further incorporation into TAG through the Kennedy pathway. When temporal and spatial expression patterns of Arabidopsis *LPCATs* were investigated using promoter fragments of *AtLPCATs* fused with the *uidA* reporter gene encoding ß-glucuronidase (GUS), differential expression patterns were observed for *AtLPCAT1* and *AtLPCAT2* [87]. Histochemical localization of GUS activity in transgenic lines showed that both promoters were active in seedling and rosette leaves. During seed development, *AtLPCAT1* promoter activity was detected at a later developmental stage, whereas *AtLPCAT2* promoter was active throughout seed development; it was also particularly strong in pollen grains. We have identified ten isotigs of *PfLPCAT1* and one isotig of *PfLPCAT2* in the lesquerella seed transcriptome, and found high sequence identity between lesquerella and Arabidopsis *LPCATs* (Table 5, Figure 7A). Our expression profiling data indicate that *PfLPACTs* are also differentially expressed (Figure 7B, 7C). During seed development, *PfLPCAT1* showed a linear-rise throughout most stages up to 42 DAP, and then a sharp drop at 49 DAP when seeds enter the desiccation stage. The *PfLPCAT1* temporal pattern resembles *AtLPCAT1*. Little expression of *PfLPCAT1* was detected in leaf, stem, root and flower bud (Figure 7B). Unlike *PfLPCAT1*, *PfLPCAT2* showed high expression during early (14-21) DAP stages of seed development, medium-high levels from 28-35 DAP, and then suddenly became undetectable and extremely low at late stages 42 and 49 DAP, respectively (Figure 7C). In leaf, stem and root, moderate expression of *PfLPCAT2* was detected (Figure 7C). In flower bud, *PfLPCAT2* was expressed highest among all organs investigated. (Figure 7C). The overall expression profile of *PfLPCAT2* is similar to that of *AtLPCAT2* based on promoter activity analysis [83] and information available at the Arabidopsis eFP browser [75]. Our results revealed that both *PfLPCATs* were expressed during seed development but with opposite trends. Thus, it is likely that both *PfLPCATs* contribute to TAG metabolism in lesquerella seeds. *PfLPCAT2* was highly expressed in flower bud, suggesting its involvement in TAG synthesis in pollen.

**Figure 8:**
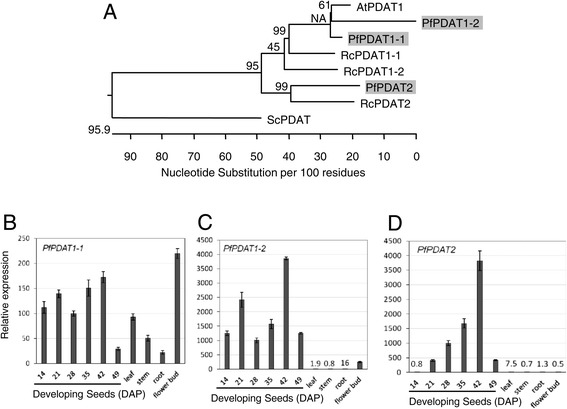
**Characterization of three** ***PfPDATs.*** **(A)** Phylogenetic tree of PDATs from *Physaria fendleri* (Pf)*,* Arabidopsis (At)*,* castor bean (Rc), *Saccharomyces cerevisiae* (Sc). *PfPDAT1-1, PfPDAT1-2, PfPDAT2, AtPDAT1*(At5g13640), *AtPDAT2*(At3g44830), *RcPDAT1-1* (HM807520), *RcPDAT1-2* (HM807521), *RcPDAT2* (HM807522), *ScPDAT* (NM_001183185). The tree was constructed as described in Figure 5B. **(B-D)** Expression of *PfPDAT1-1, PfPDAT1-2, and PfPDAT2* in developing seeds and major organs determined by qPCR*.*Abbreviated names for the genes are described in Figure 3. Each data point represents the mean ± SD of three replicates.

PDAT was identified and characterized as an acyl-CoA-independent transacylase that transfers the fatty acid at the *sn*-2 position in PC to the *sn*-3 position of DAG, thus synthesizing TAG [37]. Two PDATs were found from Arabidopsis, *PDAT1* (At5g13640) and *PDAT2* (At3g44830) [89]. *AtPDAT1* is ubiquitously expressed [75,90], but *AtPDAT2* transcripts are predominantly found in seed [75]. Together with *AtDGAT1, AtPDAT1* has been shown to determine the amount of TAG synthesis in pollen grains and developing seeds, whereas *AtPDAT2* does not play a substantial role in TAG synthesis, although it is highly expressed during seed development [91]. Based on homology searches with Arabidopsis *PDATs*, we identified a total of 28 isotigs of *PfPDAT* transcripts in the lesquerella seed transcriptome. Detailed comparison among 28 isotigs led to discovery of *PfPDAT1-1*, *PfPDAT1-2*, and *PfPDAT2* (Table 5). *PfPDAT1-2* is slightly closer to *AtPDAT1* than *PfPDAT1-1* (Table 5, Figure 8A). Sequence alignments revealed that the 3′-UTR’s of *PfPDAT1-1* and *PfPDAT1-2* were distinct (Additional file 1: Figure S4). Gene expression analysis revealed that *PfPDAT1-1* was ubiquitously expressed in developing seeds at all stages and in other organs examined (Figure 8B), similar to the expression pattern of *AtPDAT1*. Expression levels of *PfPDAT1-2* were about 100-fold higher in developing seeds compared with *PfPDAT1-1*, but were negligible in leaf, stem and root (Figure 8C). In flower bud, both *PfPDAT1-1* and *PfPDAT1-2* isoforms were highly expressed at equivalent (Figure 8B, 8C). The temporal expression of *PfPDAT2* showed an linear rise during seed development up to 42 DAP, and then a drop to a low level at the latest stage 49 DAP. The highest level at 42 DAP is comparable to that of *PfPDAT1-2*. In leaf, stem, root and flower bud, the expression level of *PfPDAT2* was negligible (Figure 8D). The role of PDAT in HFAs synthesis has been well studied in castor by two groups [50,51], who both found that castor *PDATs* were comprised of three members, *RcPDAT1-1* [51] (same as *RcPDAT1B* [50]), *RcPDAT1-2* [51] (same as *RcPDAT1A* [50]) and *RcPDAT2*. Gene expression analysis indicated that *RcPDAT1-1/RcPDAT1B* and *RcPDAT2* had profiles similar to those of *AtPDAT1* and *AtPDAT2*, respectively, [50,51]. However, *RcPDAT1-2/RcPDAT1A* was highly expressed in developing castor seeds, and this isoform was not found in Arabidopsis. Since castor accumulates 90% HFA in seed TAG, it has been proposed that the enzyme encoded by *RcPDAT1-2/RcPDAT1A* could be important for HFA-TAG synthesis. Indeed both groups demonstrated that *RcPDAT1-2/RcPDAT1A* facilitated the transfer of HFAs from PC into TAG in transgenic Arabidopsis. Unlike castor oil which has over 70% of TAGs esterified with 18:1-OH in all three positions [92], lesquerella TAGs contain 55-60% 20:1-OH, and majority of them are esterified in *sn*-1 and *sn*-3 positions [3]. The lack of HFA at the *sn*-2 of TAG could be explained by LPATs in lesquerella discriminating 20:1-OH substrate. Recent studies of transgenic *Camelina* expressing *RcFAH12* and *PfKCS3* suggested the mechanism of 20:1-OH in TAG assembly mainly utilizing Kennedy pathway in lesquerella [93]. Therefore, PfPDATs are unlikely major enzymes channeling HFA into TAGs. The roles of PfPDATs remain to be determined. The gene sequence and expression profile of PfPDATs identified in this study provide critical information for future investigation on the enzymatic activity and substrate specificity in acyl-lipid metabolism in lesquerella.

Fatty acids at the *sn*-1 and *sn*-2 positions in PC can be directly transferred to TAG through DAG converted by PDCT. The PDCT enzyme, encoded in Arabidopsis by the Reduced Oleate Desaturation1 (*ROD1*) gene, catalyzes the inter-conversion between DAG and PC by phosphocholine head group exchange [36]. In castor, the 18:1-OH is produced by the hydroxylation of 18:1 that is esterified to the *sn*-2 position of PC [39]. Since PDCT catalyzes the shuffling of acyl groups between PC and DAG, it provides a mechanism of making HFA-DAG from HFA-PC, thus the HFA-DAG can be subsequently converted to HFA-TAG. A castor PDCT enzyme gene was isolated and co-expressed in a transgenic Arabidopsis line carrying RcFAH12. The doubly transformed line had increases of 17-23% in seed HFA content [35]. The authors noted that co-expression of *AtPDCT* did not increase HFA in transgenic Arabidopsis, indicating that *RcPDCT* had evolved to effectively convert HFA-PC to HFA-DAG [35]. In the lesquerella seed transcriptome, we have identified one isotig25038 showing high homology with Arabidopsis *PDCT* and have designated it *PfPDCT*. The *PfPDCT* sequence shares 89% and 73% identify with *AtPDCT* and *RcPDCT*, respectively (Additional file 1: Figure S5). Gene expression analysis indicated that *PfPDCT* is expressed ubiquitously in developing seeds and other organs examined (Figure 4D). Among most samples, the expression levels ranged from 100-243 relative copy number, with exception of the seed sample at 42 DAP and the root sample, which had levels of about 365 and 11, respectively. A similar expression profile for *AtPDCT* was reported [36]. It is known that the *sn*-2 position of TAG in lesquerella consists almost all of C18 unsaturated acyl groups including 18:1, 18:2 and 18:3 [3]. Thus PfPDCT would not be a major enzyme involved in channeling HFA into lesquerella TAGs. It is possible that PfPDCT contributes FA flux through PC-derived DAG in TAG assembly in lesquerella. However, based on the expression profile of *PfPDCT*, it is likely that PfPDCT plays a general house-keeping function in lesquerella acyl-lipid metabolism.

## Conclusions

Lesquerella is valued for its unusual HFA in seeds. Deep sequencing of cDNAs from developing lesquerella seeds was carried out to identify candidate genes that are associated with the synthesis of seed TAG enriched with HFA. A total of 26,995 unique genes from 651 mega-base raw sequences were assembled and 74% of them (19,861) had homology with known genes. The vast majority (95%, 18,868) of the matched genes showed highest homology to Arabidopsis genes, confirming the close relationship between the two species. The results provide a molecular basis for translating findings from the model plant Arabidopsis to facilitate lesquerella crop improvement. Genes involved in the synthesis of FA and TAG were identified and annotated by detailed sequence alignments. We have identified nearly all of the known genes for *de novo* FA biosynthesis and export from the plastid, and all of the known genes for TAG assembly in ER. In addition, we characterized the temporal and spatial expression profiles of 15 key genes in TAG metabolism using quantitative RT-PCR. The sequence and gene expression data presented in this study will serve as a useful resource for future research on lesquerella and other oilseed crops and promote their development into safe sources of HFA.

## Methods

### Plant material and general growth conditions

The *P. fendleri* seeds, WCL-LY2 [4], were kindly provided by Dr. David Dierig (USDA-ARS, National Center for Genetic Resources Preservation, Fort Collins, Colorado 80521, USA). Plants were germinated and grown in a greenhouse at temperatures between 28°C (day) and 18°C (night), with supplemental metal halide lighting to provide a 15-h-day length (1000 to 1250 μmol m^-2^ s^-1^). Mature flowers were individually hand-pollinated and tagged, and the tagging dates were recorded as 0 day after pollination (0 DAP). Developing seeds at 7, 14, 21, 28, 30, 35, 42 and 49 DAP were frozen immediately in liquid nitrogen after harvest and stored at -80°C. Leaf and stem tissues were obtained from mature plants, and root tissue was obtained from 2 month old seedlings cultured in half-strength MS liquid medium [94]. Our flower sample consists of mature flower buds. Once the tissues were harvested, they were frozen immediately in liquid nitrogen and stored at -80°C.

### RNA preparation, cDNA library construction and sequencing

Total RNA was extracted from developing seeds using TRIzol Reagent (Invitrogen, Carlsbad, CA). RNA pellets were dissolved in RNAse-free water, quantified by NanoDrop ND-1000 spectrophotometer (NanoDrop Technologies, Inc., Wilmington, DE). RNA quality was checked by 2% agarose gel electrophoresis. Total RNA from the 30 DAP sample was used for preparing an mRNA sample and subsequently constructing of a cDNA library using Illumina ® TruSeq™ RNA Sample Preparation Kit (Illumina Inc., San Diego, CA ). In brief, the mRNA was purified using poly-T oligo attached to magnetic beads. Following purification, the mRNA was fragmented into small pieces using divalent cations under elevated temperature. The cleaved RNA fragments were copied into first strand cDNA using reverse transcriptase and random primers. This was followed by second strand cDNA synthesis using DNA Polymerase I and RNase H. These cDNA fragments then went through an end repair process, the addition of a single ‘A’ base, and then ligation of the adapters. The products were then purified and enriched with PCR to create the final cDNA library. The cDNA library was sequenced on a GS FLX Titanium sequencing platform (Roche, Branford, CT).

### Assembly and gene annotation

High quality sequence reads from seed libraries were assembled into isotigs and singletons using GS De Novo Assembler (v 2.6) software with the option for de novo transcriptome assembly. Clean singletons were processed to obtain high quality clean sequences, SeqClean was used to trim adapter sequences and Lucy (version 1.20p) was used to remove low quality sequences and those < 100 bp. As a result, total 21,912 singletons were generated.

To annotate the detected genes, a BLASTx search against the NCBI non-redundant protein (NR) database (http://www.ncbi.nlm.nih.gov/refseq/) was performed with an E-value threshold of less than 10^-3^. NR annotation was used to obtain GO annotation of genes according to molecular function, biological process and cellular component ontologies (http://www.geneontology.org/).

### Quantitative RT-PCR

Total RNA was reverse transcribed using the QuantiTect Reverse Transcription Kit (QIAGEN, Valencia, CA) according to manufacturer’s directions. The resulted cDNA samples were used in PCR reactions. Standard PCR amplification reactions were carried out in a volume of 25 μL containing 20 ng of cDNA, 0.5 μM each of forward and reverse primers and 1× SYBR Select Master Mix, CFX (Applied Biosystems) using a 7500 Fast Real-Time PCR system (Applied Biosystems) and standard default thermal cycling conditions [initial step, 95°C for 10 min for polymerase activation; 40 cylces of PCR, 95°C, 15 s for melting, 60°C, 1 min for annealing and extending; and dissociation step set by the system software]. Putative oligonucleotide primers were designed using Primer Express, version 3 software (Applied Biosystems). To ensure maximum specificity and efficiency during quantitative PCR, putative primer pairs were further tested for linearity of response by constructing standard curves on five or six serial 10-fold dilutions. The templates used for the standard curve analysis were mixed cDNAs from developing seeds, leaf and flower samples with a starting concentration of 20 ng/μL. For each primer set, standard curves were analyzed independently at least three times, and standard curves repeatedly showing correlation coefficients of 0.99 or higher and PCR efficiencies between 83 and 107% were accepted. PCR product specificity was confirmed by melting-curve analysis and by electrophoresis on 4% agarose gel to ensure that PCR reactions were free of primer dimers and non-specific amplicons. Information on primer pairs and their PCR efficiencies is listed in Additional file 2: Table S1. The method of Pfaffl [95] was applied to calculate comparative expression levels between samples. The *P. fendleri* 18S gene was used as internal reference to normalize the relative amount of RNAs for all samples. For each selected gene, triplicate sets of PCR reaction samples including the 18S controls, and duplicate negative controls (reaction samples without cDNA templates), were prepared and run in a 96-well plate. The average C_T_ from 28 DAP measurements were calibrated as 100 or 1000 copy numbers, and the relative copy numbers of a gene were averaged over triplicates. The PCR experiments were repeated three times for each plate to ensure that similar results could be obtained.

## Availability of supporting data

The sequence raw data from this study have been submitted to the NCBI Sequence Read Archive (SRA) http://www.ncbi.nlm.nih.gov/bioproject/260225) under the BioProject ID PRJNA260225.

## Additional files

Additional file 1: Figure S1. Protein sequence alignment among PfDGAT1-1 (isotig11157), PfDGAT1-2 (isotig11156), AtDGAT1 (At2g19450), and RcDGAT1 (XP_002514132). Black shading indicates identical amino acids. Gray shading indicates similar amino acids and no shading indicates dissimilar amino acids. Dashes indicate gaps in alignment. **Figure S2.** Protein sequence alignment among PfDGAT2 (isotig19956), AtDGAT2 (At3g51520), and RcDGAT2 (XP_002528531). **Figure S3.** Protein sequence alignment among PfDGAT3 (isotig08903), AtDGAT3 (At1g48300), and AhDGAT3 (AAX62735). **Figure S4.** C-terminal coding region of nucleotide sequence alignment between PfPDAT1-1 (isotig08780) and PfPDAT1-2 (isotig08781). Locations of qRT-PCR primer sequences for each gene are indicated with red arrows. **Figure S5.** A. Phylogenetic tree showing relatedness among PfPDCT (isotig25038) (shaded), AtPDCT (At3g15820) RcPDCT (XP_002517643). B. Amino acid sequence alignments among the same three sequences. PfPDCT is partial cDNA sequence. Additional file 2: Table S1. The lesquerella gene primers used in the qPCR analysis.
