# Supplementary material for: Identification of hydroxy fatty acid and triacylglycerol metabolism-related genes in lesquerella through seed transcriptome analysis
Source: BMC Genomics. 2015 Mar 24;16(1):230. doi: 10.1186/s12864-015-1413-8 (PMC4381405; doi:10.1186/s12864-015-1413-8)
Supplement: Additional file 1: Figure S1. — Protein sequence alignment among PfDGAT1-1 (isotig11157), PfDGAT1-2 (isotig11156), AtDGAT1 (At2g19450), and RcDGAT1 (XP_002514132). Black shading indicates identical amino acids. Gray shading indicates similar amino acids and no shading indicates dissimilar amino acids. Dashes indicate gaps in alignment. Figure S2. Protein sequence alignment among PfDGAT2 (isotig19956), AtDGAT2 (At3g51520), and RcDGAT2 (XP_002528531). Figure S3. Protein sequence alignment among PfDGAT3 (isotig08903), AtDGAT3 (At1g48300), and AhDGAT3 (AAX62735). Figure S4. C-terminal coding region of nucleotide sequence alignment between PfPDAT1-1 (isotig08780) and PfPDAT1-2 (isotig08781). Locations of qRT-PCR primer sequences for each gene are indicated with red arrows. Figure S5. A. Phylogenetic tree showing relatedness among PfPDCT (isotig25038) (shaded), AtPDCT (At3g15820) RcPDCT (XP_002517643). B. Amino acid sequence alignments among the same three sequences. PfPDCT is partial cDNA sequence. [file 12864_2015_1413_MOESM1_ESM.pptx]

## Slide 1
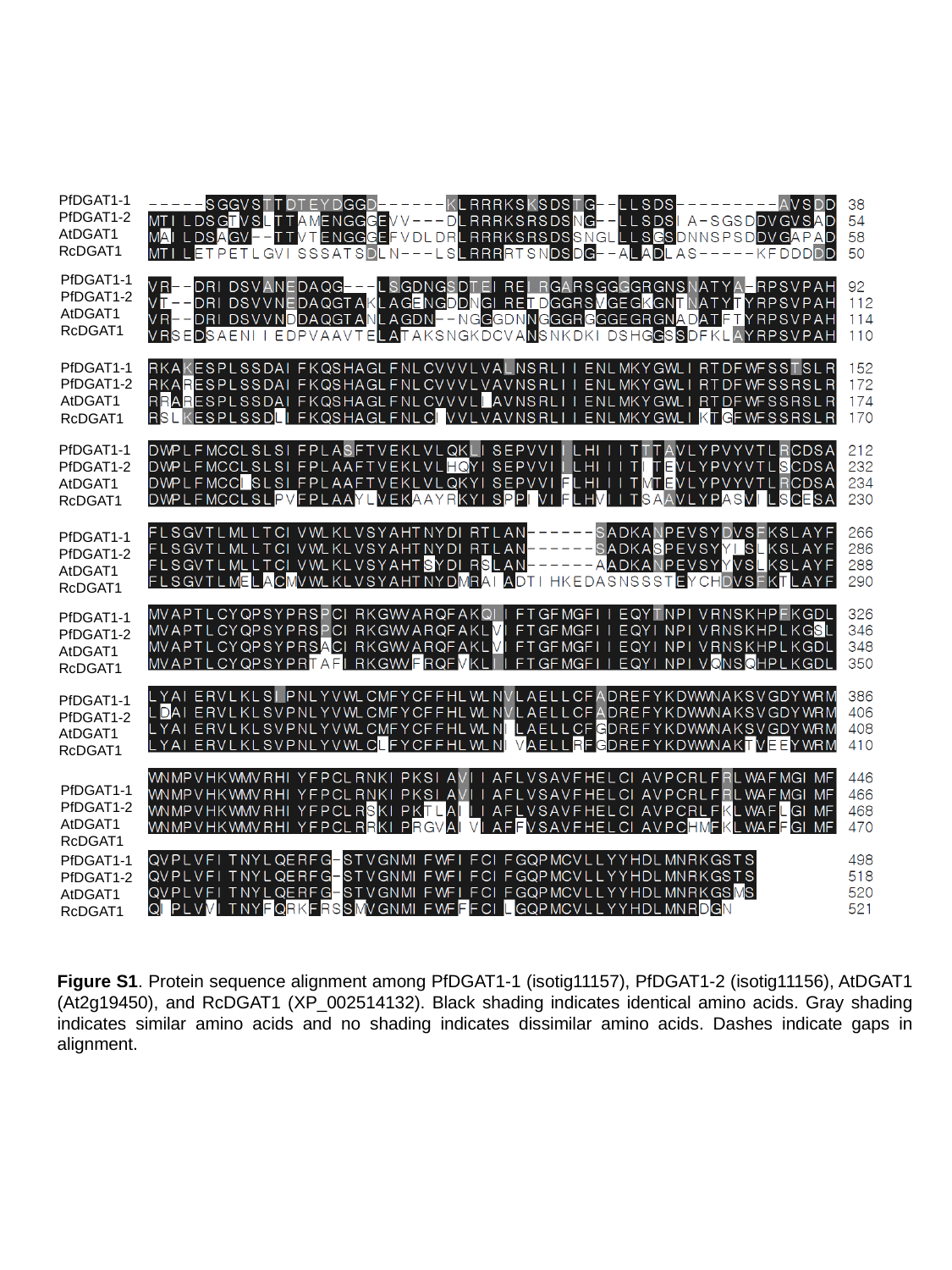

PfDGAT1-1
PfDGAT1-2
AtDGAT1
RcDGAT1
PfDGAT1-1
PfDGAT1-2
AtDGAT1
RcDGAT1
PfDGAT1-1
PfDGAT1-2
AtDGAT1
RcDGAT1
PfDGAT1-1
PfDGAT1-2
AtDGAT1
RcDGAT1
PfDGAT1-1
PfDGAT1-2
AtDGAT1
RcDGAT1
PfDGAT1-1
PfDGAT1-2
AtDGAT1
RcDGAT1
PfDGAT1-1
PfDGAT1-2
AtDGAT1
RcDGAT1
PfDGAT1-1
PfDGAT1-2
AtDGAT1
RcDGAT1
PfDGAT1-1
PfDGAT1-2
AtDGAT1
RcDGAT1
Figure S1. Protein sequence alignment among PfDGAT1-1 (isotig11157), PfDGAT1-2 (isotig11156), AtDGAT1 (At2g19450), and RcDGAT1 (XP_002514132). Black shading indicates identical amino acids. Gray shading indicates similar amino acids and no shading indicates dissimilar amino acids. Dashes indicate gaps in alignment.

## Slide 2
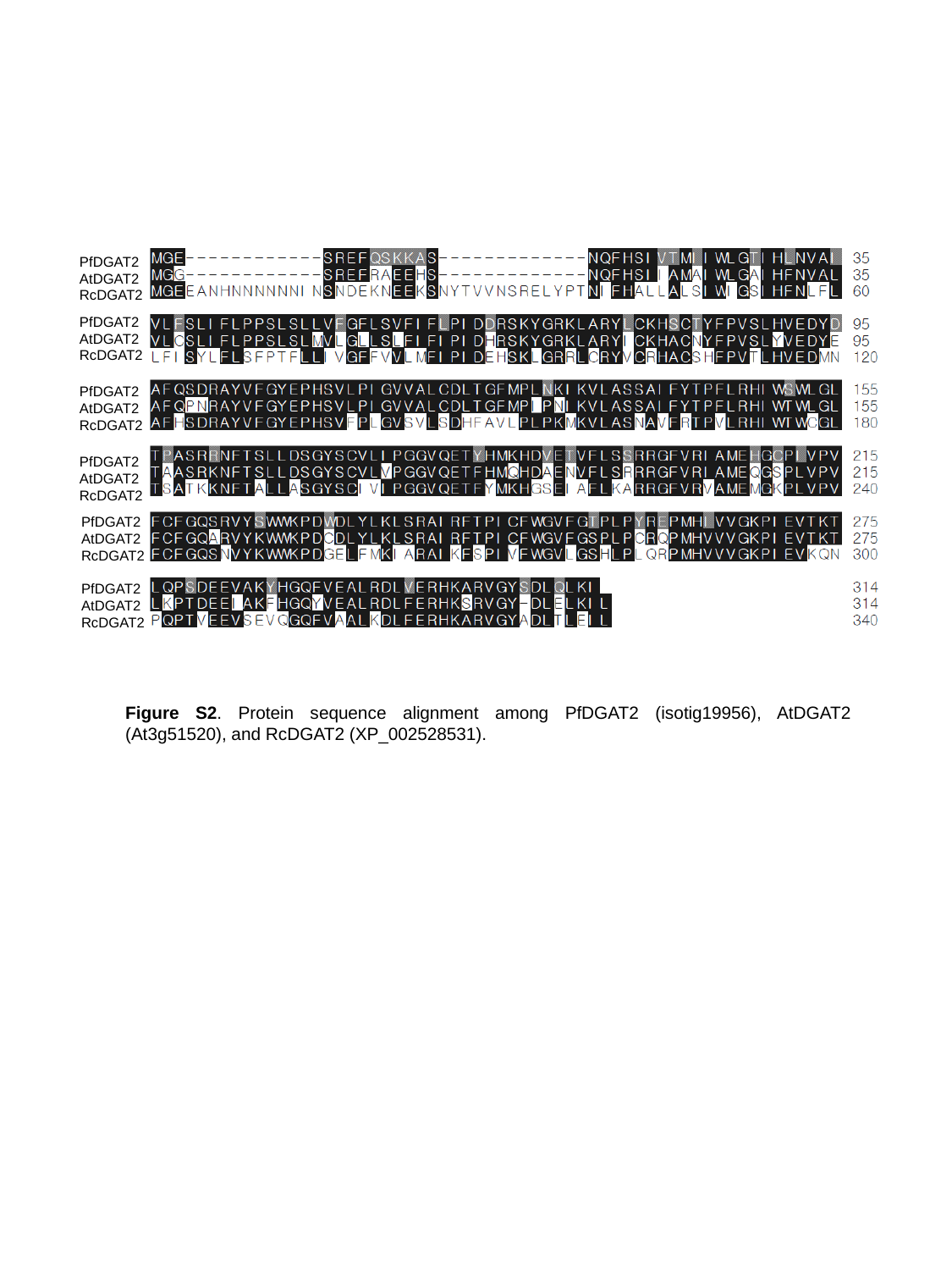

PfDGAT2
AtDGAT2
RcDGAT2
PfDGAT2
AtDGAT2
RcDGAT2
PfDGAT2
AtDGAT2
RcDGAT2
PfDGAT2
AtDGAT2
RcDGAT2
PfDGAT2
AtDGAT2
RcDGAT2
PfDGAT2
AtDGAT2
RcDGAT2
Figure S2. Protein sequence alignment among PfDGAT2 (isotig19956), AtDGAT2 (At3g51520), and RcDGAT2 (XP_002528531).

## Slide 3
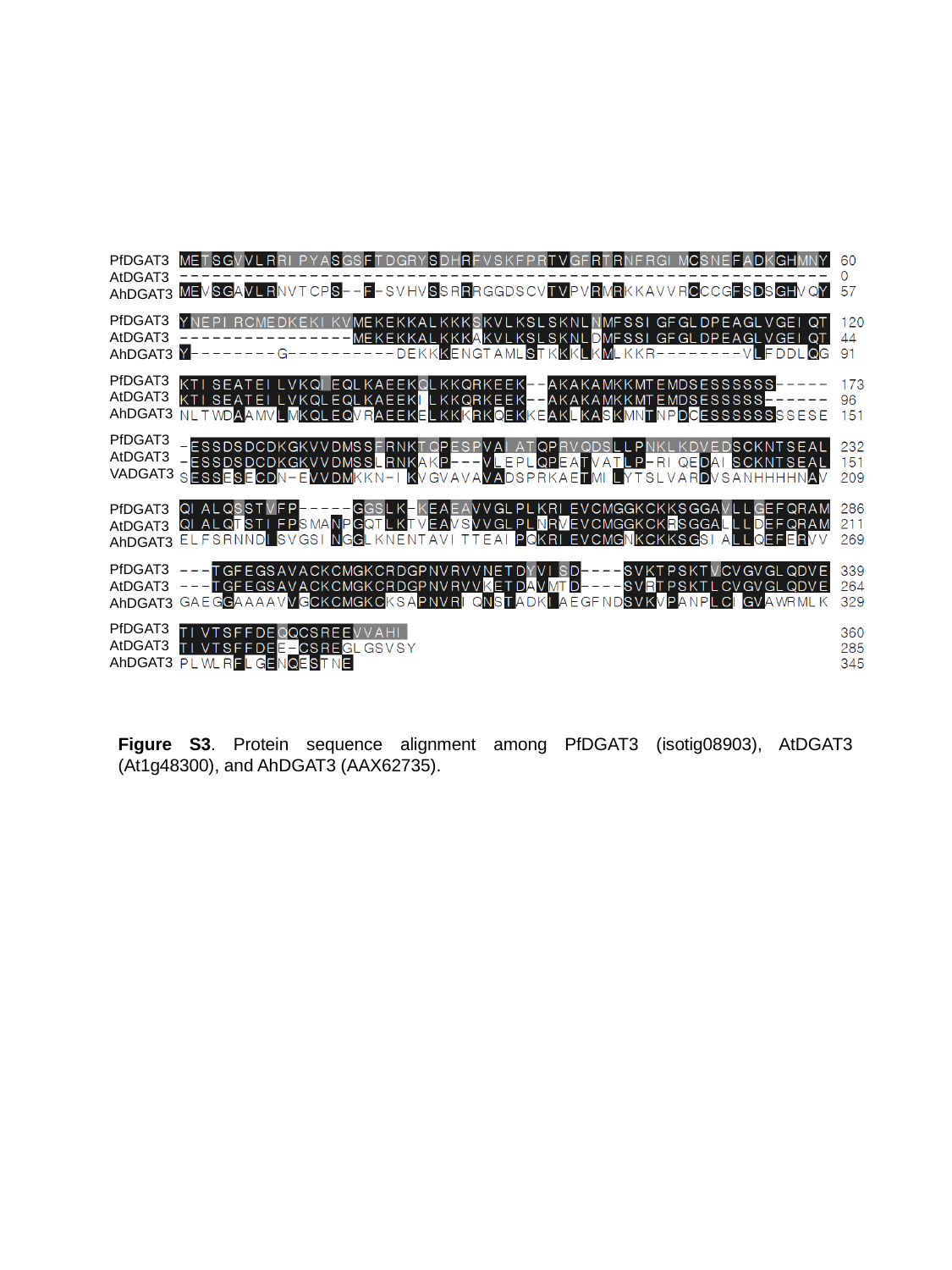

PfDGAT3
AtDGAT3
AhDGAT3
PfDGAT3
AtDGAT3
AhDGAT3
PfDGAT3
AtDGAT3
AhDGAT3
PfDGAT3
AtDGAT3
VADGAT3
PfDGAT3
AtDGAT3
AhDGAT3
PfDGAT3
AtDGAT3
AhDGAT3
PfDGAT3
AtDGAT3
AhDGAT3
Figure S3. Protein sequence alignment among PfDGAT3 (isotig08903), AtDGAT3 (At1g48300), and AhDGAT3 (AAX62735).

## Slide 4
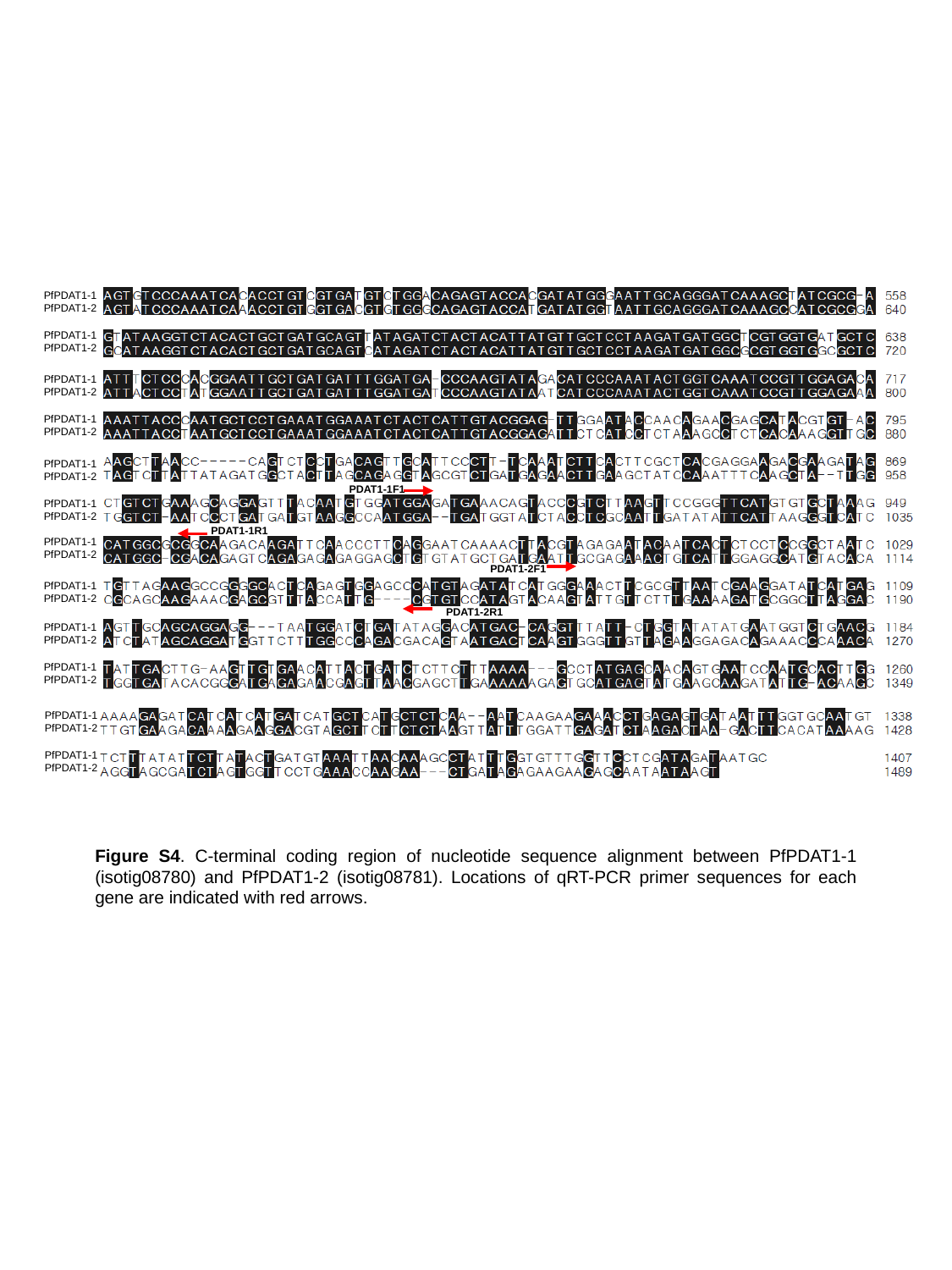

PfPDAT1-1
PfPDAT1-2
PfPDAT1-1
PfPDAT1-2
PfPDAT1-1
PfPDAT1-2
PfPDAT1-1
PfPDAT1-2
PfPDAT1-1
PfPDAT1-2
PDAT1-1F1
PfPDAT1-1
PfPDAT1-2
PDAT1-1R1
PfPDAT1-1
PfPDAT1-2
PDAT1-2F1
PfPDAT1-1
PfPDAT1-2
PDAT1-2R1
PfPDAT1-1
PfPDAT1-2
PfPDAT1-1
PfPDAT1-2
PfPDAT1-1
PfPDAT1-2
PfPDAT1-1
PfPDAT1-2
Figure S4. C-terminal coding region of nucleotide sequence alignment between PfPDAT1-1 (isotig08780) and PfPDAT1-2 (isotig08781). Locations of qRT-PCR primer sequences for each gene are indicated with red arrows.

## Slide 5
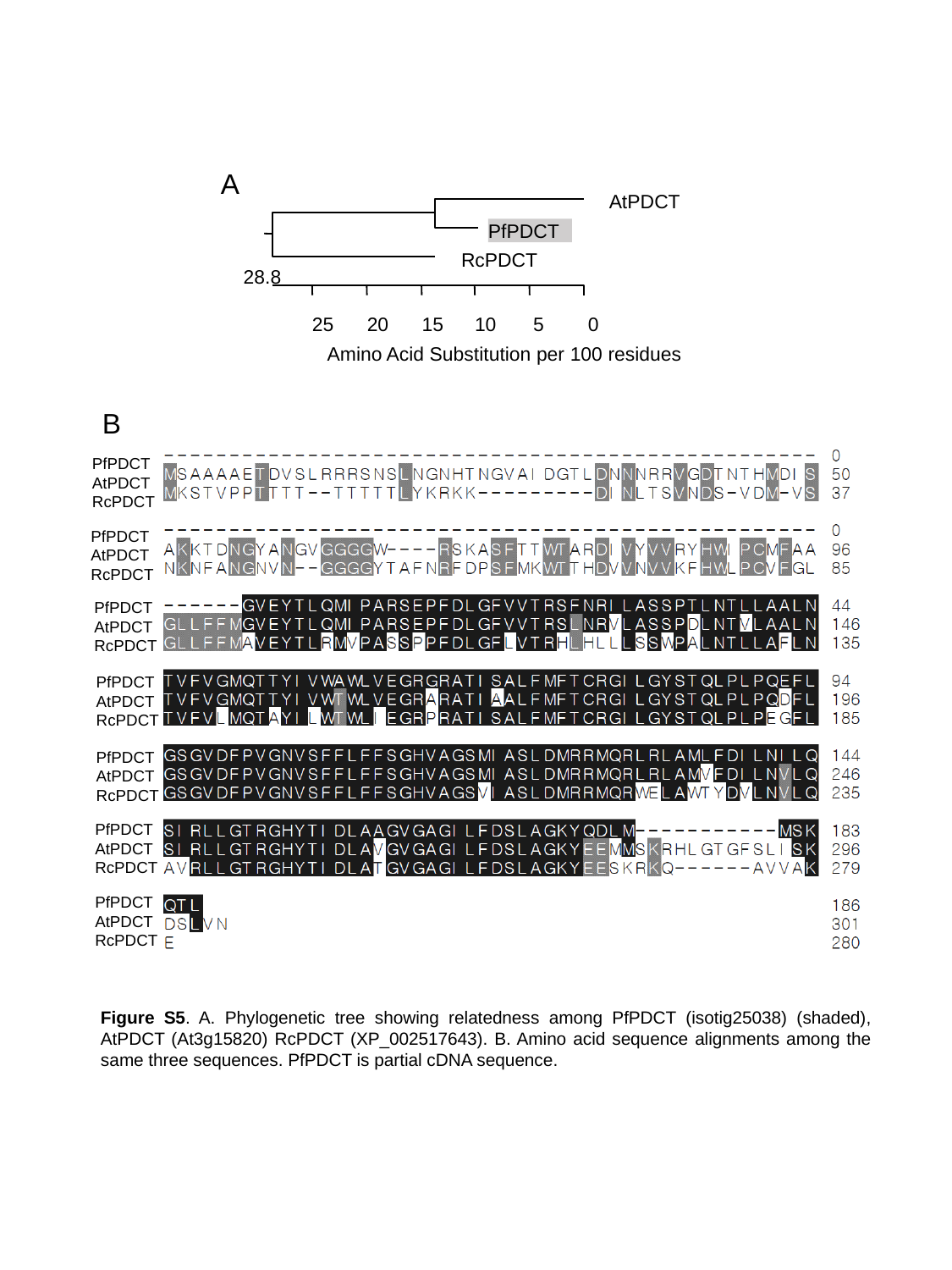

A
AtPDCT
PfPDCT
RcPDCT
28.8
25
20
15
10
5
0
Amino Acid Substitution per 100 residues
B
PfPDCT
AtPDCT
RcPDCT
PfPDCT
AtPDCT
RcPDCT
PfPDCT
AtPDCT
RcPDCT
PfPDCT
AtPDCT
RcPDCT
PfPDCT
AtPDCT
RcPDCT
PfPDCT
AtPDCT
RcPDCT
PfPDCT
AtPDCT
RcPDCT
Figure S5. A. Phylogenetic tree showing relatedness among PfPDCT (isotig25038) (shaded), AtPDCT (At3g15820) RcPDCT (XP_002517643). B. Amino acid sequence alignments among the same three sequences. PfPDCT is partial cDNA sequence.
