# Supplementary material for: Identification of hydroxy fatty acid and triacylglycerol metabolism-related genes in lesquerella through seed transcriptome analysis
Source: BMC Genomics. 2015 Mar 24;16(1):230. doi: 10.1186/s12864-015-1413-8 (PMC4381405; doi:10.1186/s12864-015-1413-8)
Supplement: Additional file 2: Table S1. — The lesquerella gene primers used in the qPCR analysis. [file 12864_2015_1413_MOESM2_ESM.docx]

**Table S1.** Information of lesquerella gene primers used in the qPCR analysis

| Abbreviated name | Forward primer, Reverse primer | PCR efficiency |
| --- | --- | --- |
| *FAD2* | CGTGTCAAGGCTGTGTCCTAAC  AGCGTGGTGGCCACATTC | 1.96 |
| *FAD3-1* | CCCTTTTCTGGGCCATCTTC  TGTCCGAGAAACTCCCATGTC | 1.99 |
| *FAD3-2* | TGCTTTCGCCGCTGTTC  CCTTGGGCGACCCAGTAAA | 1.83 |
| *GPAT9* | TGGGCTGTTGTGTGTGAAGTG  CCCTGTTTCACCTGGCCTTA | 2.01 |
| *LPAT2* | CCAGAGGAGGCCGCATACT  TGGAGGGAACTCGCTTTACAG | 1.90 |
| *DGAT1-1* | GCTCGACCTTCGGTTCCA  GATTGCGTCGGAGCTGAGA | 1.95 |
| *DGAT1-2* | CGTCGACGGAAATCGAGATC  GGAACCGGATGCAATGGA | 1.96 |
| *DGAT2* | GCGCATAGCCATGGAACAT  ACCCGTGACTGCCCAAAG | 1.91 |
| *DGAT3* | AAGCCACAGCAGAACCTTCAA  GCTGTGTTATTGGGAGAGTTTCAA | 1.94 |
| *LPCAT1* | CGCCGTTCTCCGTTTCCT  CGCCAAGCGAATGAAACAG | 1.95 |
| *LPCAT2* | GTGCTGTTTGGCATGGACTCT  CGATCATCAATGCCGACTGT | 1.96 |
| *PDAT1-1* | GGAGATGAAACAGTACCCGTCTTAA  GCCGCGCCATGCTTTA | 1.95 |
| *PDAT1-2* | ATTGCGAGAAACTGTCATTGGA  CGCAATGGTAAACGCTCGTT | 1.96 |
| *PDAT2* | GGTACTGGTTCCAGGGATTGTG  TTCAGCGCAAGGCTTACCTT | 2.04 |
| *PDCT1* | GTTAGTGGAGGGACGAGGAAGA  CCGCGACAAGTGAACATGAA | 1.92 |
| *18S* (control) | GAGAAACGGCTACCACATCCA  CCGTGTCAGGATTGGGTAATTT | 2.05 |
